# Amino acids as wetting agents: surface translocation by *Porphyromonas gingivalis*

**DOI:** 10.1038/s41396-019-0360-9

**Published:** 2019-02-19

**Authors:** M. Fata Moradali, Shirin Ghods, Thomas E. Angelini, Mary Ellen Davey

**Affiliations:** 1 Department of Oral Biology, College of Dentistry, University of Florida, Gainesville, FL 32610 USA; 2 Department of Mechanical & Aerospace Engineering, University of Florida, Gainesville, FL 32610 USA

**Keywords:** Bacteriology, Biofilms

## Abstract

Our understanding of how oral microbiota adapt in response to changes in their surroundings remains limited. This is particularly true of the slow-growing anaerobes that persist below the gum line. Here, we report that the oral anaerobe *Porphyromonas gingivalis* strain 381 can surface translocate when sandwiched between two surfaces. We show that during movement, this bacterium alters its metabolism, specifically side products of arginine utilization including citrulline and ornithine accumulated in the translocating cells; while arginine, N-acetyl-arginine, and the polyamine putrescine, which is produced from arginine were consumed. In addition, our results indicate that movement requires modification of the surrounding environment via proteolysis, cell dispersion, cell-on-cell rolling, and sub-diffusive cell-driven motility. We also show that production of fimbriae and fimbriae-associated proteins; as well as the regulation of contact-dependent growth inhibition genes, which are known to be involved in self-nonself discrimination, and the type IX secretion system are central to surface translocation. These studies provide a first glimpse into *P. gingivalis* motility and its relationship to ecological variables.

## Introduction

*Porphyromonas gingivalis* is strongly implicated in the onset and progression of periodontitis, a chronic inflammatory disease of the gingival tissues with systemic impact on human health [1–4]. This metabolically atypical bacterium persists in the subgingival crevice adjacent to the epithelium where the microbial burden is diverse and a continuous flow of gingival crevicular fluid (a serum exudate containing high levels of albumin) and microbial metabolites govern existing ecological dynamics. Due to its ability to orchestrate dysbiotic inflammation and disrupt host-microbial homeostasis even at low abundance, current models describe *P. gingivalis* as a keystone pathogen [5, 6]. Yet, given that this anaerobe can colonize the gingival sulcus in the absence of periodontal disease in an otherwise healthy mouth [7–10], and that it does not induce disease in germ free mice [11, 12] it follows that its pathogenic potential is likely both strain and context dependent [13]. Importantly, although it is well documented that *P. gingivalis* is asaccharolytic and highly proteolytic and that it utilizes protein substrates as a main source for energy production and proliferation [14–17]; the in situ physiology and metabolic adaptation of *P. gingivali*s, as well as its potential exploitative, competitive, or mutualistic interactions with the surrounding ecosystem remain largely unknown. Here, we studied two *P. gingivali*s type strains (381 and W83) that have distinct cell surface properties and colonization phenotypes. Strain 381 is a highly fimbriated strain, does not produce a capsule, and forms a robust biofilm. In contrast, strain W83 is encapsulated, does not express Mfa1 fimbriae and has only sparse FimA fimbriae, and is deficient in biofilm formation [18]. Both strains have a functional type IX secretion system (T9SS), which is essential for the secretion of a variety of proteases and protein modifying enzymes.

While other Bacteroidetes species display a T9SS-mediated gliding motility that is readily apparent on the surface of an agar plate, surface translocation by *P. gingivalis* has never been observed [19, 20]. Here, by using an anaerobic chamber slide system and time-lapse microscopy, we show that *P. gingivalis* (strain 381) can display cell dispersion and surface translocation; and establish new colonization sites. Using a combination of transcriptomic, genomic, and metabolomic approaches, we identified governing genes and cellular pathways utilized during surface translocation versus biofilm formation. Overall, our data indicate that during migration, *P. gingivalis* produces a complex metabolome, while a variety of metabolites are consumed. From an ecological perspective, our studies discovered that this keystone pathogen can forage and disperse, key ecological processes that not only support access to new sites and resource pools, but also mechanisms that could potentially affect oral microbiome structure and function as a system.

## Materials and methods

### Bacterial strains, growth conditions, and chemicals

*P. gingivalis* strain 381 (Dr. Kuramitsu, State University of Buffalo, Buffalo, NY), *P. gingivalis* strain W83 (Christian Mouton, Laval University, Quebec City, Quebec, Canada), *Escherichia coli* strain DH5-α (New England BioLabs GmbH), *Corynebacterium matruchotii* ATCC14266 and *Streptococcus gordonii* DL-1, and *Prevotella intermedia* strain 17 were used in this study. Trypticase Soy Broth (Becton, Dickinson and Company, Franklin Lakes, NJ, USA) supplemented with 5 μg/ml hemin and 1 μg/ml menadione (TSBHK) was used for cultivation of all Bacteroidetes species. TSBHK supplemented with 5% defibrinated sheep blood (BAPHK), or Brain Heart Infusion Broth without sucrose (BD Biosciences) supplemented with 5 μg/ml hemin and 1 μg/ml menadione (BHIHK) were used for cultivation and surface translocation analysis as indicated. Desired concentrations of agar or agarose were generated with Bacto™ Agar. For BSA-supplemented assays, HyClone™ Bovine Serum Albumin (GE Healthcare Life Sciences) was applied. The BS buffer contained 14 mM Na_2_HPO_4_, 10 mM KCl, 10 mM MgCl_2_, pH 7.3. *P. gingivalis* strains were incubated at 37 °C in a COY anaerobic chamber (Coy Lab Products, Grass Lake, MI, USA) under an atmosphere of 5% hydrogen, 10% carbon dioxide, and 85% nitrogen. Enzymes for genetic manipulations and cloning were purchased from New England BioLabs, Ipswich, MA, USA, and all chemicals were purchased from Sigma-Aldrich unless otherwise indicated. Fluorescent polystyrene microspheres (fluorospheres), 1 µm in diameter, were purchased from Thermo Scientific.

### Construction of mutants and in trans complementation

Deletions of *sprA* (PGN_0832), *mfa5* (PGN_0291) and *fimC* (PGN_0183) and *in-trans* complementations were performed using the NEBuilder HiFi DNA assembly cloning kit (New England BioLabs) as described previously [21]. Details are provided in Supplemental Materials and Methods and primers used to generate linear fragments are listed in Supplemental Table 1.

### Chamber slide, microscopic time-lapse, and SEM/Cryo-SEM

Designing chamber slides (0.5 mmH × 1.5 mmW × 3.0 mmL) (Supplemental Fig. 1A) for imaging at the interface of agar medium and coverslip under anaerobic conditions is fully described in Supplemental Materials and Methods. Phase contrast microscopy and time-lapse imaging were performed using an inverted Nikon Eclipse Ti microscope system (Nikon, Tokyo, Japan). Surface translocation was monitored and recorded every 5 min for 7–10 days and every 15 msec for 2–3 min for recording fast movements. SEM imaging was conducted at the Electron Microscopy core of Interdisciplinary Center for Biotechnology Research (ICBR), University of Florida (See supplemental Materials and Methods). The LIVE/DEAD BacLight bacterial viability kit (Molecular Probes) was applied for viability test.

### Classification of motility with cell tracking

**Fig. 1:**
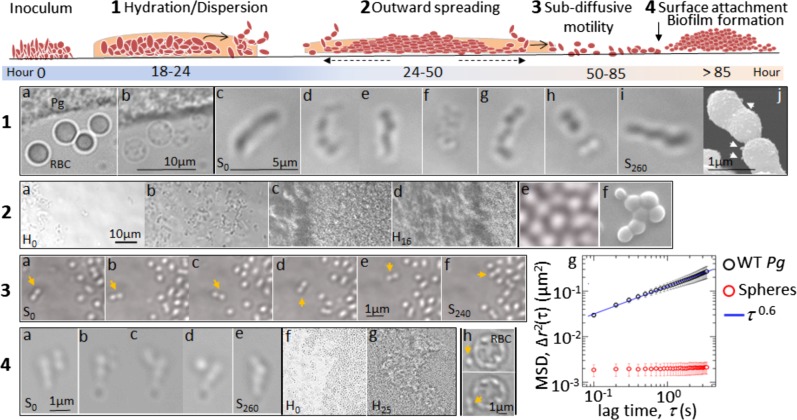
Time-lapse microscopic recordings of surface translocation by *P. gingivalis* (Pg) strain 381. Schematic illustration on top represents sequential stages recorded for more than 85-hours incubation of stabbed cells in the chamber slide. Frame 1 **a**, **b** show hydration stage and degeneration of red blood cells (RBC) and **c**–**i** represents tracking of cell-on-cell rolling and displacement within a pseudofilament over 260 sec (see supplemental video 1); **j** is SEM image of cell-to-cell contact in a pseudofilaments and arrows point out bottom-like surface protrusions. Frame 2 **a**, **b** show wriggling and forward movement of pseudofilaments followed by **c**, **d** outward spreading of stabbed colony and surface translocation over 16 h (see supplemental video 2); **e** represents continuous cell-to-cell contacts of neighboring cells during surface translocation and **f** cryo-SEM of contacting cells. Frame 3 **a**–**f** 240-second tracking of sub-diffusive cell-driven motility by cells recorded at approximately 85 h after incubation (see supplemental video 3); a paired cell marked with arrows represents migration on surface as sandwiched between agar medium and coverslip. The plot of trajectories is presented in Supplemental Fig. 2A, B **g** shows the graph of mean-squared-displacement (MSD) vs time (second) for individual cells between 50 and 85 h of surface translocation in chamber slides (black circles) and passive fluorospheres (red circles). Frame 4 **a**–**e** show 260-second tracking of topical cells rolling on a pseudofilaments attached to surface via bottom cell; **f**, **g** show surface attachment and biofilm formation and development over 25 h (see supplemental video 4); **h** shows *P. gingivalis* rolling and displacement (marked with arrow) on a red blood cell (RBC)

We performed this analysis on time-lapse recordings of cell motility observed for individual cells between 50 and 85 h of surface translocation in chamber slides (200–300 cells per sample in *N* = 3 different samples). To ensure that all measured tracks followed individual cells and could not inadvertently hop between neighboring cells, tracks exhibiting step sizes between frames larger than the single cell diameter were discarded. Additionally, only tracks longer than 70 frames (7 sec) were analyzed. Because acquisition delay time between frames of 0.1 sec is too short to track the majority of cells, which move more than their own diameter between successive frames, this analysis must be considered as proof of cell-driven motility based on the tracking of the slowest 10–20% of cells within the field of view. Supplemental Fig. 2 shows the plots of cell motion quantifications. For classifying the motion of diffusing particles and motile cells, we computed the mean-squared-displacement as the most commonly employed statistical average, given by $\Delta r^2\left( \tau \right) = \left\langle {\left( {{\bf{r}}\left( {t + \tau } \right) - {\bf{r}}\left( t \right)} \right)^2} \right\rangle$, where **r**(t) is the location of a cell at time *t* in the X–Y plane, *τ* is the delay time between pairs of location measurements for a single cell, and angle brackets denote an average over time and the ensemble of tracked cells [22, 23]. See Supplemental Materials and Methods for full description and Fig. 1, frame 3 g (error bars correspond to standard error). Computing a probability density function of all measured *α*’s, we find a slightly skewed distribution that peaks at approximately *α* = 0.6 (Supplemental Fig. 2). To test whether the motion of *P. gingivalis* was due to transient fluid-flow associated with sample preparation, the same measurements were performed on passive fluorospheres deposited in the sample chamber in the same way as the cells. No fluorosphere motion was observed (Fig. 1, frame 3g). Here, we calculated the mean speed at both the shortest and the longest time-scales (See Supplemental Materials and Methods). In our case, since the MSD scales like τ ^0.6^, the root-mean-square (rms) speed should scale like τ ^−0.7^ as shown in Supplemental Fig. 2.

### Biofilm formation assay

The biofilm assay was performed as previously described [24, 25] with slight modifications as TSBHK medium was used for growth and solubilization of 0.1% safranin was performed using 90% ethanol containing 1% sodium dodecyl sulfate (SDS) for 30 min.

### Transcriptomic analysis, computational analysis of RNA-seq data, and bioinformatic analyses

To generate plates with surface translocating cells, the inoculum (from BAPHK agar grown for 4-days) was stabbed through the soft agar (0.3% agar) layer until cells resided at the bottom on the polystyrene plate surface. A similar set of BAPHK plates, but with solid agar (1.5% agar), were inoculated on the surface providing multiple small colonies as solid agar controls. At least three replicates were used for each set of samples. Plates were incubated between 24 and 30 h, which is the time before spreading cells formed biofilms and turned brownish. This time point corresponds to the timing in our time-lapse analysis when the early to mid-stages of surface translocation was observed.

RNA extraction for RNA-seq and qRT-PCR analyses were performed in the anaerobic chamber to avoid aerobic stress using Direct-zol^™^ RNA MiniPrep Kit (Zymo Research) with slight modification (See Supplemental Material and Methods). Downstream processing for preparing RNA samples was conducted at Gene Expression & Genotyping core of Interdisciplinary Center for Biotechnology Research (ICBR), University of Florida (See supplemental Materials and Methods). Sequencing was performed on the Illumina^®^ HiSeq^®^ 3000 system instrument using the clustering and sequencing reagents provided by Illumina^®^. Paired-end, 2 × 100 cycles runs required the adding together of reagents from the 150 cycles and the 50 cycles kits (Cat# FC-410-1002, FC-410-1001, and PE-410-1001). Sequencing reactions were set up using 5 μl of the library (2.5 nM).

The program ‘Rockhopper’ [26] was used for aligning sequencing reads to the genome references (i.e., *P. gingivalis* 381, ATCC 33277, and W83), assembling transcripts, identifying their boundaries and constructing transcriptome maps, quantifying transcript abundance, data normalizing, and testing for differential gene expression (*q*-value < 0.01). Degust web tool [27] was also applied for visualizing differential gene expression. Identification of differentially expressed genes and determining operonic organization and possible cognate metabolic and non-metabolic cellular processes were conducted using various bioinformatics databases mainly including KEGG [28], BioCyc [29, 30], and the National Center for Biotechnology Information (NCBI) databases (https://www.ncbi.nlm.nih.gov).

### Untargeted global metabolomic analysis

Preparation of samples for metabolomics analysis and the number of replicates were similar to the procedure for transcriptomic analysis, except two types of media including soft agar BAPHK and BHIHK were examined. Within 24 to 30 h after incubation, the agar layer was removed, and samples were harvested from the surface of polystyrene plates using phosphate-buffered saline (or PBS, pH 7.2). The same procedure was undertaken for control preparation, except plates were not inoculated with bacterial cells. Cells were removed from collected samples and collected samples were freeze-dried, weighted, and subjected to further purification using a solvent system consisting of acetonitrile: methanol: acetone (8:1:1). Metabolomic analysis was conducted in Southeast Center for Integrated Metabolomics (SECIM) Center at the University of Florida (See Supplemental Materials and Methods). The open-source software MZmine [31] was used to identify features and deisotopes was applied for comparative and statistical analyses. Additional details are provided in Supplemental Materials and Methods.

### Statistical analysis

Statistical analysis for metabolomics data was based on univariate analysis by ANOVA using the MetaboAnalyst software [32]. It was performed separately on the positive and negative ion data from all data sets. For other data, the Shapiro-Wilk test was used to evaluate the normality of distribution of the data. It was indicated that the data were normally distributed (*P*-value < 0.05), therefore, the results were statistically analyzed by analysis of variance (ANOVA) followed by post hoc Tukey’s honestly significant difference (HSD) test for pairwise comparisons using XLSTAT statistical add-in software for Microsoft Excel 14.0. All experiments were conducted using at least three repetitions, and data are presented as means ± standard error. Differences in the data were considered significant when the probability value was <5.0% (*P*-value < 0.05).

## Results

### Colony expansion at the subsurface of soft agar by *P. gingivalis*

In a chronic periodontal lesion, *P. gingivalis* persists within a complex microbial community in the subgingival crevice, between the tooth surface and the adjacent epithelium, which extends 4–12 mm in depth [33, 34]. Since *P. gingivalis* harbors all the major protein components constituting a type IX secretion system and this secretion system is linked to gliding motility, we sought to better simulate subgingival conditions and thereby test the hypothesis that surface translocation is conditional and strain dependent in *P. gingivalis*. To this end, two strains; 381 (a highly fimbriated robust biofilm-forming strain) and W83 (an encapsulated strain that is deficient in attachment and biofilm formation) were tested. Although these strains are profoundly different in their surface properties and biofilm phenotype, both strains grow at the same rate and harness the T9SS. To compare and contrast their ability to migrate, the strains were stabbed in soft agar medium (BAPHK medium containing 0.3% agar) and incubated anaerobically. Within 24 h, colonies expanded from the site of inoculation at the interface between the agar layer and the polystyrene Petri dish (Supplemental Fig. 1B). Within 4 days, expanding colonies of strain 381, but not W83, were shaped with concentric, circular zones of growth (or zonal patterns) with many small colonies (satellite microcolonies) appearing around the periphery of the biofilm (Supplemental Fig. 1C–E). Microscopic examination showed many individual cells separated from the expanding colony, indicating that *P. gingivalis* cells of strain 381 might utilize a specific type of dispersal mechanism (Supplemental Fig. 1F). In contrast, while strain W83 displayed colony expansion, it lacked the ability to attach to the surface of the polystyrene Petri dish, form zonal patterns, or satellite microcolonies (Supplemental Fig. 1C). Our working hypothesis is that structural differences in cell surface properties of the two strains result in a dissimilar degree of surface attachment and cell dispersion. These observations motivated us to perform a series of anaerobic time-lapse studies and Cryo-SEM imaging for detailed analysis of colony expansion or dispersion by *P. gingivalis* strains.

### Surface translocation by strain 381 involves modification of the subsurface, dispersal, cell-on-cell rolling, and sub-diffusive motility

To analyze *P. gingivalis* behavior at the subsurface of soft agar via time-lapse microscopy, the chamber slide system was used to track cells inside the anaerobic incubator for 10 days (Supplemental Fig. 1A). By placing cells of strain 381 and W83 under soft BHI and BAPHK (0.3% agar) and time-lapse imaging, we were able to record the differences in colony expansion and colonization by the two strains. Only strain 381 displayed a complex motility behavior and a full cycle of growth from initial colonization to cell dispersion/surface translocation and to re-colonization of the coverslip, resulting in distal biofilm formation. In contrast, W83 demonstrated a simple and passive colony expansion from inoculation site by the force of cell proliferation without demonstrating cell dispersion, surface translocation, and re-colonization. The chronology of time-lapse recordings of strain 381 demonstrated that within 18–24 h after inoculation, numerous cells dispersed from the stabbed colony which was concomitant with wetting and clearing of the peripheral area of inoculation site. This clearing of the coverslip appeared around the initial colony as a distinct zone. Indeed, the hydrating stage (proteolysis and reduction in surface tension) correlated with hydrolysis and clearing of the red blood cells in the agar (Fig. 1, frame 1a, b). Cryo-SEM imaging during the early stages of migration revealed a hollow zone that formed around the inoculum, indicating the initial stages corresponds to medium digestion (Supplemental Fig. 1G). Within 24 to 50 h, dispersed cells proliferated and organized in cellular chains, here designated as pseudofilaments (Fig. 1, frame 1c–I; Supplemental video 1). Interestingly, the majority of *P. gingivalis* cells within pseudofilaments were coccoid shaped and in pairs, and these cells demonstrated constant rolling capability on top of neighboring cells, moving in different directions (Fig. 1, frame 1c–j). Cooperative and collective cell-on-cell rolling induced a specific movement to the pseudofilament structures, resulting in forward displacements within the hydrated area, resembling a wriggling motion (Fig. 1, frame 2a, b; Supplemental video 1). During the period between 50 and 85 h, cells that had proliferated at the site of inoculation demonstrated outward spreading along with frequent and direct cell-to-cell contact (Fig. 1, frame 2c–f; Supplemental video 2). During this time, pseudofilaments either disassembled to individual cells with the capability of individual surface translocation (Fig. 1, frame 3a–f and supplemental video 3) or the cells attached to the coverslip via bottom cell(s) while the cell(s) on top continued to roll on neighboring cells (Fig. 1, frame 4a–e). Later in this stage, some cells lost their movement and attached to the surface. These colonizers developed a new biofilm structure over the next 24 h (Fig. 1, frame 4 f, g; Supplemental video 4). In contrast, similar analysis with strain W83 demonstrated a simple and passive colony expansion from inoculation site, the formation of pseudofilaments, cell dispersion, surface attachment, and new colonizers were not seen over the course of experiment for this strain. These dissimilarities could arise from the lack of fimbrial structures on the cell surface of strain W83, as well as the production of a capsule. We concluded that strain W83 is non-motile.

Time-lapse recordings of individual cells (strain 381) demonstrating surface translocation (Fig. 1, frame 3a–f; Supplemental video 3) were subjected to computational characterization to classify the observed motility. The mean-squared-displacement, Δ*r*^2^(*τ*), for motile cells was computed from the measured tracks of individual cells in each sample (see material and methods). The ensemble and sample averaged measurement of Δ*r*^2^(*τ*) indicated that *P. gingivalis* moved in a sub-diffusive manner, rising approximately *τ* ^0.6^ (Fig. 1, frame 3 g; Supplemental Fig. 2). This type of motion arises when stochastically moving objects are hindered by their neighbors or other obstacles in their surroundings [35]. It is important to note that sub-diffusive motility is distinct from Lévy-flight or Lévy-walking, which refers to super-diffusive random motion. Intriguingly, Lévy-flight has been used to describe the motility of *Myxobacteria*, another highly proteolytic bacterium, where a mass of swarmer cells migrates on a viscous surface that is modified by self-produced wetting agents [36, 37]. To begin to assess whether the apparent sub-diffusive motion of *P. gingivalis* was not due to transient fluid-flow associated with sample preparation, the same measurements were first performed on passive fluorospheres, deposited in the chamber slide in the same way as the cells. Tracking the fluorospheres and analyzing their motion demonstrated their immobility and being trapped between the top and bottom surfaces of the chamber slide (Fig. 1, frame 3g, Supplemental video 5). Furthermore, as discussed below, strains with mutations in surface structures proliferated on the surface but were also immobile. Thus, our data support the model that *P. gingivalis* movement at the late stage of surface migration is a form of sub-diffusive cell-driven motility. Since this motion is not ballistic, the average measured speed strongly depends on the lag-time over which distances are measured. According to our calculation for selected motile population (see Method section), the mean speed was computed as about 2 µm/s at the shortest time-scales and about 0.15 µm/s at the longest time-scales measured (Supplemental Fig. 2). After 85 h incubation, individual cells which could not colonize the surface, turned into elongated and motionless cells while other cells displayed continuous back and forth movements to attach cells, forming an aggregate of disordered cells (Supplemental Fig. 1H). Live/dead staining showed that the island of misshaped and disordered cells contained numerous numbers of dead cells, while also embedding a few living cells (Supplemental Fig. 1I, J).

### Exploring the underlying mechanism of surface translocation by *P. gingivalis* strain 381

To test our initial observations in greater depth, we assessed translocation after treatment with metronidazole and we investigated the ability of other oral bacteria to surface translocate under similar conditions. The addition of metronidazole (40 µg/ml) determined that bacterial killing abolished the zone of hydration as well as cell dispersion from the inoculation site, showing that these stages require viable cells (Supplemental video 6). For comparison, we assessed three other purportedly non-motile oral bacteria including *C. matruchotii*, *S. gordonii*, and *P. intermedia* under the same growth condition. *C. matruchotii* and *S. gordonii* produced chains of rod-shaped and cocci-shaped cells, respectively, but these bacteria did not display dispersion or surface translocation (Supplemental video 7). *P. intermedia*, another oral *Bacteroides* that is highly proteolytic and has a T9SS, formed a biofilm, but it also did not demonstrate surface translocation (Supplemental video 7). Interestingly, we discovered that dispersing cells of *P. gingivalis* were capable of rolling and displacing on the surface of red blood cells (Fig. 1, frame 4h).

To begin to identify the underlying mechanism controlling motility, we used three mutant strains, specifically strains with deletions in *sprA* (or *sov*), *mfa5*, or *fimC*. The *sprA* gene encodes an outer membrane protein and a major component of the envelope spanning T9SS multiprotein complex. SprA is necessary for secretion of various virulent factors such as gingipains by *P. gingivalis* and motility adhesins by *Flavobacterium johnsoniae*, another member of the phylum Bacteroidetes [38]. *P. gingivalis* harbors other subunits of T9SS multiprotein complex, but *P. gingivalis* lacks the motility adhesins SprB and RemA characterized in *F. johnsoniae* [20, 38]. Yet, it is noteworthy that PGN_0291 (*mfa5*) of *P. gingivalis* encodes a von Willebrand factor A-containing protein which shows weak homology (21% identity) with RemA and it is secreted via T9SS, therefore we hypothesized that Mfa5 may function as a motility adhesin. Previously, it was shown that this accessory protein is incorporated into the polymerization process of minor fimbriae affecting the incorporation of other accessory subunits [39]. Lastly, we generated a *fimC* deletion mutant. FimC is an accessory component of the major fimbrial subunit, FimA, which is necessary for surface attachment and biofilm formation [40].

Our time-lapse microscopic assessment determined that the nonpigmented ∆*sprA* mutant (Supplemental Fig. 3A) did not form pseudofilaments within 80 h. Between 80 and 110 h, motionless cellular aggregates were seen around inoculation site, and we noted that the surrounding agar turned into a jelly-like texture which contained aggregates of proliferating cells. Yet, there was no observable cell movement (Supplemental video 8). Over 10 days of tracking, no cell-on-cell rolling or wriggling motion or sub-diffusive cell-driven motility was recorded for the ∆*sprA* mutant. At the later stages of incubation, many swollen and globe-shaped single cells were observed to be trapped in the modified, jelly-like medium (Supplemental video 8).

In the case of the ∆*mfa5* mutant, the zone of hydration, cell dispersal, and pseudofilament formation were recorded within 18 h, similar to the phenotype of the parent strain. Yet, the majority of pseudofilaments were found to remain in proximity to the inoculation site without any observable motion, however the cells did proliferate over time. After 80 h, motility was not observed, and many cells were scattered randomly on the coverslip, indicating the importance of Mfa5 protein in coordinating cell movements and cell-on-cell rolling (Supplemental video 8). In surface attachment and biofilm formation, deletion of *mfa5* resulted in enhanced biofilm formation, while overexpression of *mfa5* significantly reduced biofilm formation when compared with the parent strain (Supplemental Fig. 3B). Overall, these results indicate that the Mfa5 protein negatively regulates biofilm formation and has a strong association with colony dispersion and surface translocation. Similarly, the zone of hydration, cell dispersal from inoculation site, and pseudofilament formation were also seen in the ∆*fimC* mutant within 18 h, but the majority of pseudofilaments were either defective in wriggling motion or disassembled to individual diploid and motile cells (Supplemental video 9). Surprisingly, these cells actually displayed rapid surface translocation similar to social twitching or swarming motility, suggesting that *fimC* negatively regulates another type of motility (Supplemental video 9). As expected, the ∆*fimC* mutant did not show surface colonization and, therefore, no biofilm formation was recorded over 10 days of analysis. Overall, our studies show that T9SS is critical for proper modification of the environment and surface translocation, and that Mfa5 coordinates cell movements and cell-to-cell interactions; while FimC, and possibly the two other genes in this operon (FimD and FimE) negatively regulate an underlying surface translocation mechanism that requires further exploration.

From an ecological perspective, culturing of *C. matruchotii* or *S. gordonii* and *P. gingivalis* in close proximity showed that *S. gordonii*, but not *C. matruchotii* inhibited *P. gingivalis* surface translocation (Supplemental Fig. 4A, B). Although these studies are preliminary, it is evident that surface translocation by *P. gingivalis* can be impacted by other oral bacteria. Importantly, since it is well established that *P. gingivalis* and *S. gordonii* co-aggregate and grow together, our data can be interpreted as further support of their mutualistic interaction, specifically we suggest that *P. gingivalis* may stop moving and attach when it senses metabolites secreted by *S. gordonii* into the environment.

### Genetic responses by cells grown at the subsurface of soft agar are substantially different between strains

**Fig. 2:**
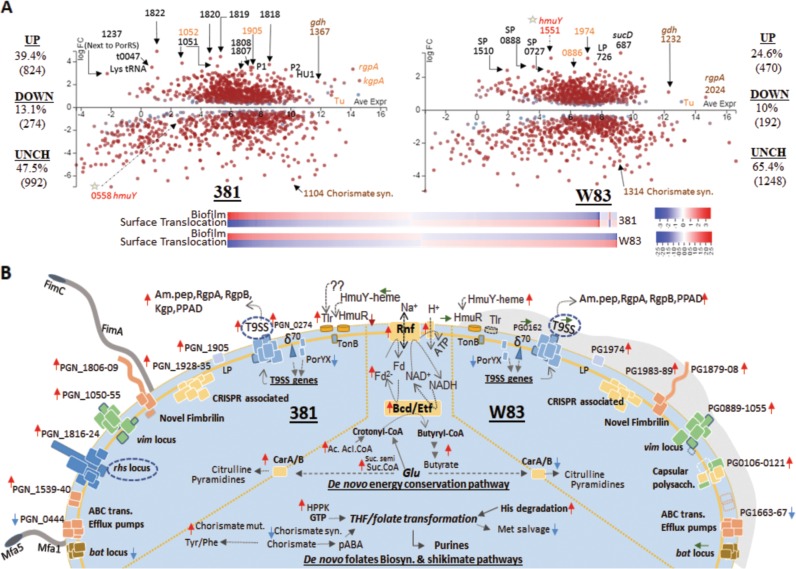
Differential gene expression (DGE) of strains 381 and W83 at 24 h when grown on the subsurface of soft agar versus the surface of solid agar. **a** The MA-plots represents DGE on a field of log2 fold change (FC) versus the average log-expression values of each gene (e.g., numerically labeled) across all samples. Different levels of genetic responses by two strains upon colony expansion are listed at both sides of plots. **b** Schematic illustration represents an alteration of various cellular pathways detected during colony expansion which was inferred from DGE data (**b**). Briefly, biological functions mediated by membrane and extracellular proteins including T9SS components and putative rearrangement hotspot (Rhs) proteins, were highly unregulated in strain 381, which demonstrated cell dispersion and surface translocation; DGE of major homologous genes and pathways of two strains did not follow the same pattern, notably for Rhs proteins and T9SS components (circled). Proteases and peptidylarginine deiminase (PPAD) which appear to be necessary for hydration were upregulated in both strains. Deduced bioenergetic pathways mediated by putative Bcd/Etf (butyryl-CoA dehydrogenase/electron transfer flavoproteins) and the Rnf (Rhodobacter nitrogen fixation) complexes and the biosynthesis of folate derivatives were upregulated in both strains during colony expansion. This change in expression was concomitant with downregulation of chorismate synthesis and methionine (Met) salvage pathways. Red, blue, and green arrows represent upregulation, downregulation and unchanged gene expressions, respectively. Gray arrows indicate biological pathways. UP: upregulation; DOWN: downregulation; UNCH: unchanged; SP: small peptides; bat: the bacteriodes aerotolerance; CRISPR: clustered regularly interspaced short palindromic repeats; Am.pep: aminopeptidase; δ^70^: the extracytoplasmic function sigma factor 70; HPPK: 7,8-Dihydro-6-hydroxymethylpterin-pyrophosphokinase; GTP: guanosine-5’-triphosphate; THF: tetrahydrofolate; CarA/B: carbamoyl phosphate synthase small subunits A/B; His: histidine; Glu: glutamic acid; Ac.Acl.coA: acetoacetyl CoA; Suc.semi: succinic semialdehyde, Suc.CoA: succinyl-CoA; Fd: ferredoxin; NAD: nicotinamide adenine dinucleotide

Transcriptomic analysis on the cells of strain 381 and W83 when grown at the subsurface of soft agar versus biofilm cells showed that approximately 51.7% (1081 genes, at 2-fold or more, *q*-value < 0.01) of the total theoretical protein coding region of the strain 381 genome and almost 34.6% of that of W83 strain genome (662 genes, at 2-fold or more, *q*-value < 0.01) were differentially expressed. Accordingly, in strain 381, the expression of 6.1% (129 genes), 0.52% (11 genes) and 0.33% (7 genes) of the genome were differentially changed by ≥10-, ≥ 20- and ≥30-fold respectively (*q*-value < 0.01; with the highest value at 114-fold), while only 5 genes (0.26% of the genome) of W83 had differential gene expression at 10-fold and more with the highest value at 18-fold. A summary of the RNA-Seq data is presented in Supplemental Table 2 and Fig. 2 and discussed in detail below.

### Genes encoding putative contact-dependent growth inhibition proteins and T9SS components are highly upregulated during surface translocation

**Fig. 3:**
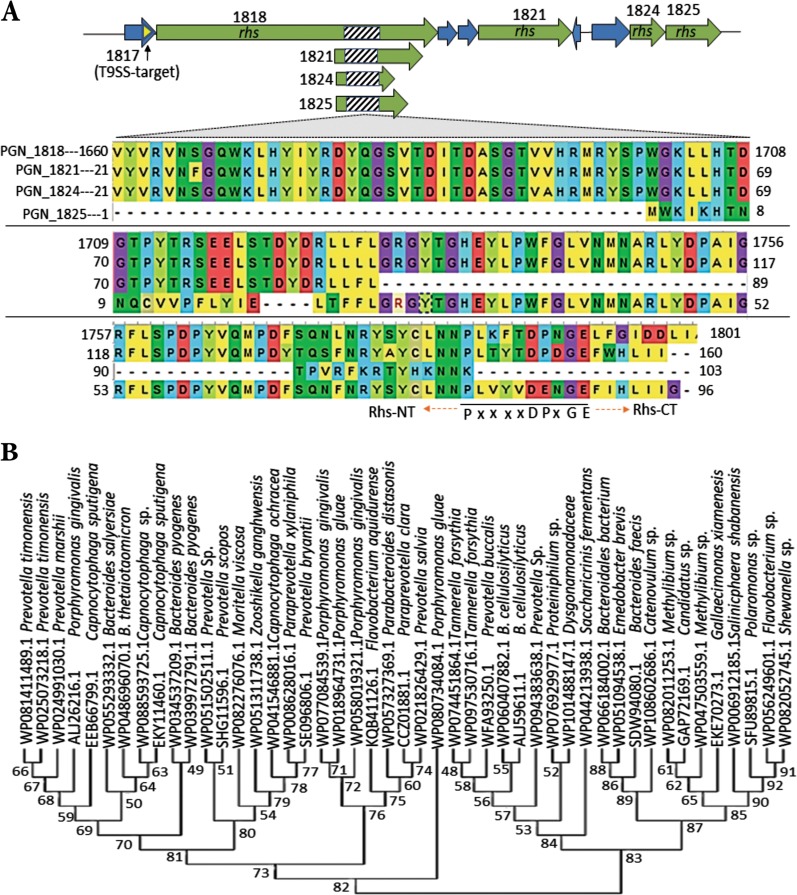
Rearrangement hotspot (Rhs) genes are the most upregulated genes in strain 381 during surface translocation. These genes are absent in the genome of strain W83. **a** Genetic organization of the different *rhs* loci in strain 381 located downstream of PGN_1817 with T9SS-cargo protein feature. Protein multiple sequence alignment by ClustalW indicated highly conserved core of these genes ending in the signature motif PxxxxDPxGE which separates it from the highly variable region. **b** Phylogenetic tree represents widely distribution of highly conserved core of *rhs* genes across Cytophaga-Flavobacterium-Bacteroides (CFB) phylum (identity ≥50%). This evolutionary tree only represents some select species. The evolutionary history was inferred by using the Maximum Likelihood method based on the Poisson correction model

Strikingly, in the translocating cells of strain 381, the genomic region encoding (PGN_1816 to PGN-1825) a putative rearrangement hotspot (Rhs) and related YD-peptide repeat (Rhs/YD-repeat) proteins were the most differentially expressed as upregulated in a range of 3.5–114-fold (*q*-value < 0.01) (Supplemental Table 2). Homologous sequences that encode full length Rhs/YD-repeat proteins were not identifiable in the genome of non-motile strain W83. The genes encoding Rhs/YD-repeat proteins are widely distributed in bacteria and have been shown to mediate contact-dependent growth inhibition (CDI) in a toxin/immunity pair manner [41, 42]. These proteins mediate growth inhibiting-function among strains of a single species as well as interspecies interactions and competitions [43]. Distribution of the genes encoding Rhs/YD-repeat proteins and their organization and biological role(s) in the Bacteroidetes have not yet been studied. Our analysis using ClustalW multiple sequence alignment of four Rhs genes on the genome of *P. gingivalis* strain 381 showed that, similar to enterobacterial Rhs proteins, these proteins share a highly conserved core sequence (134 amino acids) with a highly variable C-terminal domain, separated by the consensus sequences PxxxxDPxGE (Fig. 3a). Importantly, PGN_1817 encodes a T9SS-cargo protein located upstream of PGN_1818 encoding the largest Rhs/YD-repeat protein, suggesting a possible mechanistic linkage between *P. gingivalis* Rhs/YD-repeat proteins and T9SS, which suggests intriguing similarity to delivery of Rhs toxins into target cells through the type V and VI systems by *Escherichia coli* and *Dickeya dadantii* [43, 44]. Using protein BLAST to analyze genome-wide protein alignments and phylogenetic analysis, we found a conserved core Rhs region of *P. gingivalis* is widely distributed in the Cytophaga-Flavobacterium-Bacteroides (CFB) phylum (Fig. 3b) including oral and gut *Bacteroides*. Furthermore, analysis of the genes encoding the T9SS complex showed that the expression of multiple genes including *sprA*, *porN/M/L/K/P/W/U* were significantly upregulated by 2.5–11-fold in the translocating cells of strain 381 (*q*-value < 0.01), yet these genes remained unchanged or had insignificant changes in strain W83 when compared with biofilm cells. It has been shown for gliding motility that the secreted T9SS-cargo proteins directly mediate surface translocation while the T9SS membrane complex not only functions in secretion, but there is data suggesting that some of the membrane proteins mediate energy transduction [45, 46]. Given the differences in expression between the two strains, we posit that strain 381 may require a higher degree of energy transduction to display a complex surface translocation than W83.

At the regulatory level, the PorYX two component system (TCS) and the extracytoplasmic function (ECF) sigma factor SigP have been shown to form a regulatory cascade with regard to expression of T9SS secretion genes [47, 48]. Interestingly, differential expression of *porY* was insignificant between two types of growth, while *porX* expression was downregulated by almost 10-fold in the translocating cells of strain 381. In contrast, the expression of SigP was upregulated (3-fold). This result is in line with the recent findings suggesting that SigP may play a major regulatory function in T9SS under certain growth conditions, instead of the PorYX system [47, 48]. Yet, it is important to note that post-translational modification (phosphorylation) of PorYX TCS, which was not examined in this study, may also be central to controlling gene expression during surface translocation.

### T9SS-cargo proteins underlie the hydration stage required for cell-on-cell rolling and sub-diffusive cell-driven motility

**Fig. 4:**
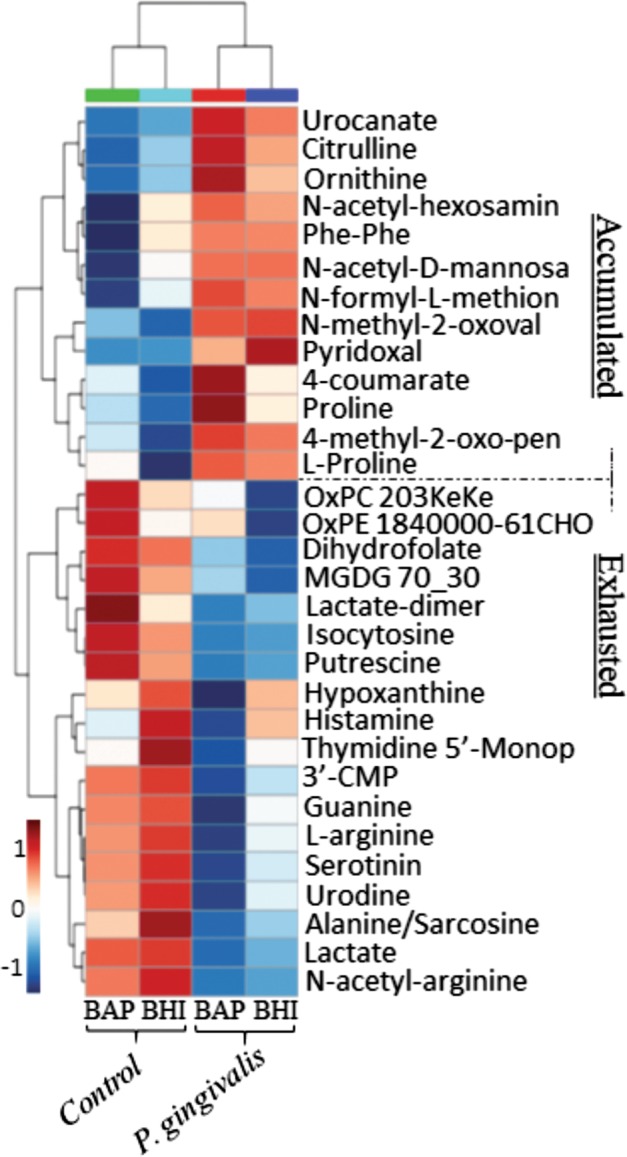
Metabolic adaptation of the translocating cells. **a** Metabolome profile provided by an untargeted global metabolomic analysis represents accumulated or exhausted metabolites in two different media during surface translocation by strain 381. Statistical analysis for metabolomics data was based on univariate analysis by ANOVA which was performed separately on the positive and negative ion data from all data sets (*p*-value <0.05)

During growth at the subsurface of soft agar, gene PGN_0508/PG1605 was upregulated by 28 and 7.1-fold (*q*-value < 0.01) in strains 381 and W83 respectively, when compared to cells grown on the solid agar surface. This gene is located adjacent to gene *PorZ*, which encodes an essential component of the T9SS. Using the Phyre2 Protein Fold Recognition Server [49], we found that gene PGN_0508/PG1605 has structural homology with cysteine proteinases and PSI-Blast analysis indicated that the protein has 35% homology with bleomycin hydrolase (BH) with the function of peptide trimming and peptide production possibly involved in releasing hygroscopic amino acids, which can act as wetting agents, as described for natural moisturizing in mammalian systems. This hydration effect has also been described for citrulline and urocanate accumulation (discussed below) [50–53]. The T9SS-cargo proteases including a zinc carboxypeptidase (PGN_0335/PG0232), a putative cysteine protease (PGN_0900/PG1427), two arginine-specific proteinases Rgp-A/B and a lysine-specific proteinase Kgp were also upregulated by up to 22-fold (*q*-value < 0.05) during surface translocation by strain 381; as was a peptidylarginine deiminase (PPAD), another T9SS- protein that converts charged arginine residues within peptides to citrulline. PPAD was upregulated by 13.4 and 5.2, in strain 381 and W83, respectively. Importantly, the transcriptomic data correlated with the metabolomic analysis of strain 381 which revealed that citrulline accumulated during surface translocation along with proline (highly hygroscopic) and uroconate (a by-product of histidine metabolism) (Fig. 4). Taken together, cumulative actions of these hydrolyzing enzymes indicate a process of natural moisturizing which may underlie certain processes during surface translocation, in particular cell-on-cell rolling, and sub-diffusive cell-driven motility by strain 381. To test this hypothesis, we treated the chamber slide with 1X Clontech’s ProteoGuard EDTA-Free Protease Inhibitor Cocktail and discovered that this treatment completely inhibited surface translocation (Supplemental Fig. 4C, D). Although we cannot rule out the inhibitive effect of this treatment on cell growth, *P. gingivalis* can efficiently grow and proliferate on the peptides available in BHI and not all proteases are inhibited by this cocktail. Our working hypothesis is that the defect in spreading resulted from a combination of reduced growth and a lack of production of wetting agents (free amino acids). In summary, our data indicate that secretion of T9SS-cargo proteins is required for proper modification of environment, which in turn, favors surface translocation.

### Strains 381 and W83 utilize different mechanisms and genetic elements for nutrient uptake when grown at the subsurface of soft agar

The HmuR protein serves as a major TonB-dependent receptor for utilizing both hemin and hemoglobin as key nutrients for growth [54–56]; where HmuR interacts with complex proton channels such as TonB-ExbB-ExbD or MotA/TolQ/ExbB located on the inner membrane to accomplish transportation [57–59]. Our RNA-Seq analysis showed that the *hmuR* gene was downregulated in strain 381, while an uncharacterized TonB-linked heme-binding receptor annotated as Tlr, without any homolog in W83, was unregulated by 14.5-fold (*q*-value < 0.01). Similarly, its cognate gene encoding heme-binding protein HmuY [54, 60, 61] was downregulated in strain 381, while it was upregulated by 15-fold (*q*-value < 0.01) in the non-motile strain W83, when compared to cells growing on the solid agar surface. Furthermore, the genes encoding putative TonB and MotA/TolQ/ExbB proton channel were respectively upregulated by 2 and 6- fold only in the translocating cells of strain 381 (*q*-value < 0.01). At the regulatory level, LuxS-dependent signaling negatively regulates *hmuR* expression in *P. gingivalis*, while it positively controls the expression of *tlr* gene [62]. Consistent with this report, our result showed that *luxS* gene was significantly upregulated in strain 381 during surface translocation; hence the inverse regulation of *hmuR* and *tlr* was likely due to LuxS-dependent signaling. LuxS is required for production of type 2 autoinducer (AI-2) which is a key molecule governing inter-species quorum sensing in a wide variety of bacteria [63]. Although a response regulator that binds AI-2 in *P. gingivalis* has not been identified, LuxS is important for controlling the expression of genes involved in the acquisition of hemin [64]. Taken together, the data show that the motile strain 381 and the non-motile W83 strain use different mechanisms and genetic elements for nutrient uptake during growth at the subsurface interface of soft agar; yet increased nutrient transport linked to iron acquisition is required in both strains.

### Bioenergetic pathways that are active during growth at the subsurface interface of soft agar

Electron bifurcation is an electron transfer mechanism used by anaerobic bacteria to maximize energy conservation. Bifurcating enzymes have been shown to be essential in diverse metabolic processes. *P. gingivalis* harbors genes predicted to be involved in energy conservation via electron bifurcation mediated by the butyryl-CoA dehydrogenase/electron transfer flavoproteins (Bcd/Etf) complex as well as the Rhodobacter nitrogen fixation (Rnf) complex [65]. The Bcd/Etf complex serves as an intermediate electron carrier between primary flavoprotein dehydrogenases and terminal acceptors such as ferredoxin or flavodoxin and crotonyl-CoA [65, 66]. Our transcriptomic analysis identified genes encoding the Bcd/Etf complex and the Rnf complex as upregulated in the cells of both strains by up to 14-fold when growing at the subsurface interface of soft agar (Supplemental Table 2). Our working model is that the production of succinyl-CoA and subsequently crotonyl-CoA via glutamate and aspartate catabolism, provides an electron acceptor for electron bifurcation as described in other bacteria [67]. This model is further supported by significant upregulation of a number of genes central to this pathway; including glutamate dehydrogenase, 2-oxoglutarate oxidoreductase, CoA-dependent succinyl-CoA reductase, NAD-dependent 4-hydroxybutyrate dehydrogenase, 4-hydroxybutyrate CoA-transferase, and 4-hydroxybutyryl-CoA dehydratase.

Importantly, a metabolic pathway for glutamate and aspartate catabolism in *P. gingivalis* has been proposed. Yoshida et al., [68, 69] showed that a CoA-dependent succinyl-CoA reductase catalyzed the conversion of succinyl-CoA in the presence of NAD(P)H to succinate semialdehyde which in turn converted to 4-hydroxybutyrate by NAD-dependent 4-hydroxybutyrate dehydrogenase [68, 69]. In this pathway, putative 4-hydroxybutyrate CoA-transferase and 4-hydroxybutyryl-CoA dehydratase can catalyze downstream steps to produce crotonyl-CoA coupling this reaction to electron bifurcation. Predicted to be associated with this pathway, is a cognate ferrodoxin gene (PGN_1752/PG1813), which was upregulated by 18- and 6-fold, respectively, in strains 381 and W83. In this process, ferredoxin can play the role of electron acceptor during formation of succinyl-CoA with NADH being the reductant [70]. Genes associated with the electron transport chain via fumarate reductase/succinate dehydrogenase flavoprotein (operon PGN_0496-98) were, in contrast downregulated in the cells growing at the subsurface of soft agar.

Lastly, as shown in Supplemental Table 2, genes predicted to be cytochrome *bd* ubiquinol oxidases (PGN_1041-42) were upregulated. Cytochrome *bd* oxidases, catalyze the two-electron oxidation of either ubiquinol or menaquinol are at the end of the respiratory chain where they couple with the Na^+^-translocating NADH:quinone oxidoreductase (Na^+^-NQR) and other dehydrogenases such as NADH-linked lactate dehydrogenase [71–74]. The Na^+^-NQR system also acts as a primary sodium pump and respiratory module at bacterial membrane that mediates electron transfer from NADH to quinone, which is coupled with the translocation of sodium ions across membrane generating a sodium motive force [75, 76] or chemiosmosis [77–84]. Here, during growth at the subsurface of soft agar, genes predicted to encode a Na^+^-NQR system were upregulated. Although the biological function of this system during this mode of growth is not clear, our preliminary assessment using 2-*n*-heptyl-4-hydroxyquinoline *N*-oxide (HQNO) (10 µg/ml) as a specific inhibitor of the Na^+^-NQR function and quinone oxidation [85, 86] followed by qRT-PCR analysis; showed that *P. gingivalis* strain 381 treated cells had a significantly reduced surface translocation (Supplemental Fig. 4E) and reduced expression of surface translocation associated genes including *sprA*, BH-like protease, and *rhs* (Supplemental Fig. 5A) and the cells were larger in size compared to the untreated samples, while they proliferated into cell aggregates (Supplemental Fig. 5B). Together, these experiments provide evidence of a shift in bioenergetic pathways in *P. gingivalis* during surface translocation with an active regulatory role of Na^+^-NQR system in biological processes and osmotic homeostasis.

### Metabolic adaptation for surface translocation by *P. gingivalis* strain 381

Using statistical analysis of the metabolic profile, we identified metabolites that are produced or exhausted due to *P. gingivalis* metabolism during surface translocation (Fig. 4). High levels of urocanate, citrulline, ornithine and proline were detected in translocating cells when compared with uninoculated subsurface media samples. Generated by a histidine ammonia-lyase, urocanate is the byproduct of histidine degradation pathway and transcriptomic analysis determined that this enzyme was upregulated. Importantly, histidine degradation also contributes to the biosynthesis of folate derivatives forming a critical pathway for the biosynthesis of amino acids and nucleic acids [87]. Our study showed that the expression of multiple cognate genes in folate biosynthesis were upregulated in the translocating cells including dihydrofolate reductase, a putative methenyltetrahydrofolate cyclohydrolase, a putative 5-formyltetrahydrofolate cyclo-ligase, and a formiminotransferase-cyclodeaminase (Fig. 2a, b). In agreement with this finding, dihydrofolate was found to be exhausted from the media (Fig. 4). In addition, production of folate derivatives can occur via chorismate- and GTP-dependent pathways [88, 89]. Interestingly, enzymes in the folate pathway including GTP cyclohydrolase I (FolE), 7,8-dihydro-6-hydroxymethylpterin-pyrophosphokinase (HPPK), and bifunctional phospho-2-dehydro-3-deoxyheptonate aldolase (DAHP)/chorismate mutase were significantly upregulated by up to 32 times in the translocating cells, while the expression of chorismate synthase was downregulated by almost 50 times followed by downregulation of methionine salvage pathway (Fig. 2, Supplemental Table 2).

The side products of arginine utilization including citrulline and ornithine also accumulated in the translocating cells, while arginine, N-acetyl-arginine, and the polyamine putrescine, which is produced from arginine were consumed. A recent study showed that, during epithelial cell colonization and abscess infection, carbamoyl phosphate synthetases contributes to the fitness of *P. gingivalis* by linking arginine and citrulline metabolism [90]. We found that the expression of these genes was increased by up to 6-fold in the translocating cells of strain 381, but they were downregulated in the non-motile W83 strain.

Lactate and lactate-dimers were found to be consumed (Fig. 4) and the transcriptome showed upregulation of lactate permease gene and predicted lactate dehydrogenases. Lactate and pyruvate and their reversible conversions by lactate dehydrogenases play vital roles in central metabolism [91]. Investigation of *P. gingivalis* colonization and surface translocation in chamber slide using a medium composed of BSAS (0.4% BSA) with lactate or pyruvate 0.5%, hemin and menadione revealed that *P. gingivalis* did not transition to the hydration stage or the cell dispersal and surface translocation stages; instead these conditions tended to promote a biofilm mode of growth. These results suggest that biofilm-to-surface translocation by *P. gingivalis* is under the control of nutritional stimuli and signals.

## Discussion

The results presented here demonstrate an as yet unreported surface motility for *P. gingivalis* historically known as a sessile, non-motile bacterium. Surface translocation by strain 381 was concomitant with high expression levels of T9SS components and Rhs genes. T9SS mediates surface gliding by other Bacteroidetes and Rhs proteins are known to mediate contact-dependent inhibition (CDI). CDI is a ubiquitous mechanism that plays a key role in competition strategies, via delivery of toxins that kills neighboring target bacteria, thereby eliminating competitors, analogous to quorum sensing for cooperative behavior such as social motility (e.g., swarming) [20, 41, 43, 92]. CDI behavior mediated by Rhs/YD-repeat proteins has the potential for ecological significance by facilitating the invasion of strains into habitats occupied by other species [93]. A core region of *P. gingivalis* Rhs modules was found to be highly conserved among members of the CFB phylum including those inhabiting the oral cavity and gut. According to our model, cumulative functions of T9SS-cargo proteases and protein modifying enzymes can result in the release of hygroscopic amino acids, which results in hydration of the surrounding environment and this hydration is required for cell-on-cell rolling and surface translocation. Since translocating *P. gingivalis* cells deploy Rhs modules, which impact interspecies competitions, it is intriguing to speculate that changes in expression of these modules may play a role in the microbial shift and dysbiotic inflammation associated with periodontitis. Importantly, biosynthesis of folate derivatives, electron bifurcation system, and quinone oxidation were found as the most utilized pathways for metabolic, energetic, and osmotic adaptions during surface translocation.

In summary, although it is remarkable that motility had not been observed previously in this bacterium, it is evident that given the correct nutritional and physical parameters, *P. gingivalis* is fully capable of complex surface migration. Moreover, although the underlying mechanisms for movement remains to be determined, it is evident that cell-cell contact, fimbrial proteins, and T9SS along with secretion of certain T9SS-cargo proteins are required. We propose that the mode of surface translocation reported here is most similar to the sliding motility reported for *Mycobacteria*, yet it appears to be distinct in mechanism [94]. Our current working model is that *P. gingivalis* surface translocation is an amalgamation of aspects of gliding and sliding motility. Moreover, we propose that the unique conditions required for migration may reflect the co-evolution of this bacterium with its human host and the complex consortium of microbes with which it exists. We propose that our findings open a new avenue for studying how commensal anaerobes adjust their lifestyle in response to nutritional and physical parameters, and the data provide a foundation for studying metabolic crosstalk in human microbiota that may be central to commensal or pathogenic status.

## Supplementary information

Supplemental Material and Methods

Supplemental Figures and Video legends

Supplemental Video 1

Supplemental Video 2

Supplemental Video 3

Supplemental Video 4

Supplemental Video 5

Supplemental Video 6

Supplemental Video 7

Supplemental Video 8

Supplemental Video 9

Supplemental Table-1

Supplemental Table-2

Supplemental figure 1

Supplemental Figure 2

Supplemental Figure 3

Supplemental Figure 4

Supplemental Figure 5
