# Supplementary material for: Amino acids as wetting agents: surface translocation by Porphyromonas gingivalis
Source: ISME J. 2019 Feb 19;13(6):1560–74. doi: 10.1038/s41396-019-0360-9 (PMC6775972; doi:10.1038/s41396-019-0360-9)
Supplement: Supplementary file 1 — Supplemental Material and Methods [file 41396_2019_360_MOESM1_ESM.docx]

**Supplemental Material and Methods**

**Construction of mutants and *in trans* complementation**

Deletions of *sprA* (PGN_0832), *mfa5* (PGN_0291) and *fimC* (PGN_0183) were generated using the NEBuilder HiFi DNA assembly cloning kit (New England BioLabs) as described in manufacturer`s protocol and explained previously (1). Primers used to generate linear fragments are listed in Supplemental Table 2. For homologous recombination, *P. gingivalis* strain 381 was transformed with generated linear fragments carrying a promoterless erythromycin resistance gene (ermF) by electroporation as previously described (2). Transformants were selected on BAPHK supplemented with antibiotic followed by further confirmation via genomic DNA isolation using The Wizard^®^ Genomic DNA Purification Kit (Promega) and amplification of upstream/downstream regions to deleted gene and DNA sequencing (Eurofins Genomics). For complementation, abovementioned assembly cloning kit was employed to insert relevant genes under the promoter of *ragA* (P*_ragA_*: 448 bp upstream of *ragA* open reading frame) on the plasmid pTCOW (3) digested with *Hind*III and *Sph*I restriction enzymes.

**Chamber slide, microscopic time-lapse, and SEM/Cryo-SEM**

Chamber slides (Supplemental Fig. 1A) were made by compressing several layers of sterilized parafilm M (Bemis) on glass slides followed by fixing of the parafilm with slight heat. The sides of the fixed parafilm were then sealed with nail polish. The center of the parafilm layer was removed with a sterile scalpel, to create a chamber (0.5mmH×1.5mmW×3.0mmL). This chamber was then filled with soft agar medium (0.3% agar). The medium was allowed to solidify, and a coverslip inoculated with a tiny dot of cells at the center was inverted and placed on chamber filled with medium and mounted with nail polish. Imaging was performed at the interface of agar medium and coverslip. Phase contrast microscopy and time-lapse imaging were performed using an inverted Nikon Eclipse Ti microscope system (Nikon, Tokyo, Japan) with Perfect Focus, automated controls (NIS-Elements, Nikon) and equipped with a motorized stage (Nikon) and an Andor Zyla 5.5 sCMOS Camera. The microscope was located inside a COY anaerobic chamber under the condition described above. Using a Nikon 100x 1.40 NA objective, surface translocation was monitored and recorded every 1 min for 7 to 10 days and every 15 msec for 2-3 min for recording fast movements.

SEM imaging was conducted at the Electron Microscopy core of Interdisciplinary Center for Biotechnology Research (ICBR), University of Florida. Samples were fixed with 2.5% glutaraldehyde, 4% paraformaldehyde in 1xPBS, pH 7.24. Cells were then washed with 1xPBS followed by deionized water and then dehydrated in a graded ethanol series (25%, 50%, 75%, 95%, 100%) followed by treatment with Hexamethyldisilazane (HMDS). Dried samples were mounted onto aluminum stubs with carbon adhesive tabs, sputter coated with Au/Pd (Denton DeskV) and imaged with Hitachi SU5000 FE-SEM (Hitachi High Technologies, America). For Cryo-SEM, samples were mounted on carbon adhesive tab which was attached to a Cyro-stage followed by plunge freezing in liquid nitrogen. A frozen sample was placed into the Quorum PP3010T Cryo-SEM Preparation System (Quorum Technologies) to be sublimed for 10 minutes, then sputter coated with gold/palladium for 45 seconds. Next, the sample was placed inside the SEM chamber onto a cold stage at -195 degrees to image with the abovementioned SEM equipment. The LIVE/DEAD BacLight bacterial viability kit (Molecular Probes) was applied for viability test.

**Classification of Motility with Cell Tracking**

We performed this analysis on time-lapse recordings of cell motility observed for individual cells between 50 and 85 hours of surface translocation in chamber slides (200-300 cells per sample in *N* = 3 different samples). To ensure that all measured tracks followed individual cells and could not inadvertently hop between neighboring cells, tracks exhibiting step sizes between frames larger than the single cell diameter were discarded. Additionally, only tracks longer than 70 frames (7 seconds) were analyzed. Because acquisition delay time between frames of 0.1 seconds is too short to track the majority of cells, which move more than their own diameter between successive frames, this analysis must be considered as proof of active motility based on the tracking of the slowest 10-20% of cells within the field of view. Supplemental Fig. 2 shows the plots of cell motion quantifications. For classifying the motion of diffusing particles and motile cells, we computed the mean-squared-displacement as the most commonly employed statistical average, given by
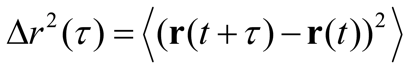
 , where **r**(t) is the location of a cell at time *t* in the X-Y plane, *τ* is the delay time between pairs of location measurements for a single cell, and angle brackets denote an average over time and the ensemble of tracked cells (4, 5). Measurements of Δ*r*^2^(*τ* ) allow the identification of different classes of motion in populations of cells or particles. For example, for purely diffusive motion in 2D corresponding to Brownian motion,
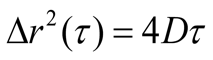
, where *D* is the single-cell diffusion coefficient. By contrast, for ballistic motion in which cells move in a straight line at a constant speed,
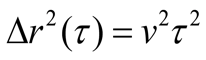
, where *v* is the particle speed. For particles moving stochastically while encountering obstacles,
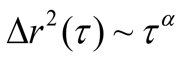
, where *α* is a dimensionless constant having a value less than 1. This type of motion is called “sub-diffusive” and is often associated with an effect called “caging” in which the motion of a particle is impeded by its neighbors. Thus, it is possible to characterize the class of motion exhibited by cells by measuring the power of *τ* to which Δ*r*^2^(*τ* ) rises.

We computed Δ*r*^2^(*τ* ) for all individual tracked cells in each sample, then computed the mean and standard deviation of Δ*r*^2^ across different cells at each delay time. Measurements for the different samples were further averaged, and a standard error was computed by combining the standard-deviations from different samples in quadrature and dividing by
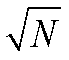
, where *N* = 3 samples (Fig. 1, frame 3g, error bars correspond to standard error). This ensemble and sample averaged measurement of Δ*r*^2^(*τ* ) indicates that *P. gingivalis* move in a sub-diffusive manner, rising approximately like * *^0.6^. To ensure that this apparent power-law did not arise from ensemble averaging single cell tracks that exhibited more complicated motion, we measured *α* at all *τ* ’s for each cell by computing the logarithmic derivative of each single-cell Δ*r*^2^(*τ* ), which is given by
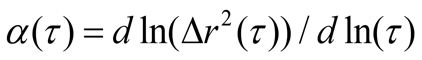
. Computing a probability density function of all measured *α*’s, we find a slightly skewed distribution that peaks at approximately *α* = 0.6 (Supplemental Fig. 2). To test whether the motion of *P. gingivalis* was driven by active cell motility and not transient fluid-flow associated with sample preparation, the same measurements were performed on passive fluorospheres deposited in the sample chamber in the same way as the cells. No fluorosphere motion was observed (Fig. 1, frame 3g). Accordingly, our measurements for speed calculation represent averages among this population as abovementioned. Since this motion is not ballistic, but sub-diffusive, the average measured speed will strongly depend on the lag-time over which distances are measured. Here, we calculated the mean speed at both the shortest and the longest time-scales. In our case, since the MSD scales like τ ^0.6^, the root-mean-square (rms) speed should scale like τ ^-0.7^ as shown in Supplemental Fig. 2.

**Transcriptomic analysis and RNA sequencing**

RNA extraction was performed in the anaerobic chamber to avoid aerobic stress using Direct-zol^™^ RNA MiniPrep Kit (Zymo Research) with slight modification. Briefly, RNA extraction was performed for surface translocating cells after removing soft agar layer and cells on surface of polystyrene plates were lysed using 600 µl of TRI Reagent^®^ (Zymo Research). Similar amount of reagent was applied for RNA extraction from non-motile cells growing on solid agar layer. Debris were removed from collected samples by centrifugation at 14,000 × g for 10 min. equal volume of 100% ethanol was mixed with each sample. Each mixture was loaded into a Zymo-Spin^™^ IIC column and centrifuged at 14,000 × g for 30 sec followed by washing using 400 µl of RNA wash buffer. DNA residues were completely digested and removed from columns in two rounds, each by adding 80 µl of DNase I and DNA Digestion Buffer (see Direct-zol^™^ RNA MiniPrep Kit protocol) and incubating at 30 °C for 1 h followed by centrifugation at 14,000 × g for 30 sec. Second round was followed by washing columns using 400 µl of Direct-zol^™^ RNA PreWash and 700 µl of RNA Wash buffers. Pure RNA crude was eluted twice using 15 µl of DNase/RNase-Free Water. Total amounts of RNA samples in the range of 7,000 to 25,120 ƞg were subjected to downstream preparation.

Downstream processing for preparing RNA samples was conducted at Gene Expression & Genotyping core of Interdisciplinary Center for Biotechnology Research (ICBR), University of Florida. Quality control of RNA samples was performed using Qubit^®^ 2.0 Fluorometer (ThermoFisher/Invitrogen, Grand Island, NY), and RNA quality was assessed by applying the Agilent 2100 Bioanalyzer (Agilent Technologies, Inc). RNAseq library construction was performed for samples with calculated values of 28S/18S ratio greater than 1.0 and RNA integrity number (RIN) equal or more than 7.0. Ribosomal RNAs (rRNAs) were eliminated from 600 ng of total RNA using Illumina Ribo-Zero™ Magnetic Kit for bacterial RNA according to the manufacturer’s protocol. Yielded depleted RNA were used for library construction by utilizing NEBNext^®^ Ultra^™^ RNA Library Prep Kit for Illumina^®^ (New England Biolabs) and according to the manufacturer's user guide. Briefly, 5ul of purified product was fragmented using the first strand synthesis reaction buffer mix by heating at 94 °C for 8 min followed by first strand cDNA synthesis using reverse transcriptase and random primers. Synthesis of double-stranded cDNA was conducted using the 2nd strand master mix provided in the kit. The resulting cDNA was end-repaired, dA-tailing and ligated with NEBNext adaptors. Finally, library was enriched by PCR amplification, and purified by Agencourt AMPure XP system (Beckman Coulter). For the quality control of the library and pooling, barcoded libraries were sized on the bioanalyzer and then quantitated by Qubit assay kits (Invitrogen). Typically, a 200-1000 broad library peak was observed. Quantitative PCR is used to validate the library's functionality, using the KAPA Library Quantification Kits for Illumina platforms (Kapa Biosystems, Cat# KK4824). All samples were equimolar-pooled for one lane of HiSeq 3000 2X100 cycles run. Sequencing was performed on the Illumina^®^ HiSeq^®^ 3000 system instrument using the clustering and sequencing reagents provided by Illumina^®^. Paired-end, 2x100 cycles runs required the adding together of reagents from the 150 cycles and the 50 cycles kits (Cat# FC-410-1002, FC-410-1001, and PE-410-1001). Sequencing reactions were set up using 5 μl of the library (2.5 nM).

**cDNA preparation and quantitative real time PCR (qRT-PCR)**

Sample preparation and RNA isolation was performed as described above. The reverse transcription reactions were performed by using the RNA to cDNA EcoDry Premix (Random Hexamers) kit (Clontech Laboratories, Inc.). Briefly, for each sample, 2500 ng of isolated RNA was added into 20 µl of reaction solution as final volume. Reaction was performed at 42°C for 60 min using a Bio-Rad T100^TM^ thermocycler followed by stopping it at 70°C for 10 min. For relative quantification of desired genes qPCR assays was conducted in a 20 µl total volume containing 1 µL of 1:10 diluted cDNA, 0.5 µM of each primer (Supplemental Table 2), 6 µl of PCR grade water and 10 µl of 2×iQ SYBR Green Supermix. Amplification and detection of product were performed using the CFX96 Touch™ Real-Time PCR Detection System (Bio-Rad) and cycling condition was applied as the following: 95°C for 3 min, and then 39 cycles of 95°C for 20 sec, 55°C for 20 sec and 72°C for 20 sec and fluorescence was detected after each cycle. In each experiment, the target and control samples were amplified in the same plate and conducted in triplicate and normalized internally using simultaneously the average cycle quantification (Cq) of the reference gene. To confirm specificity of the amplified products, automated melting curve analysis was performed.

**Bioinformatic analyses**

Identification of differentially expressed genes, their operonic organization and possible cognate metabolic and non-metabolic cellular processes were conducted using various bioinformatics databases mainly including KEGG (8), BioCyc (9, 10), and National Center for Biotechnology Information (NCBI) databases (<https://www.ncbi.nlm.nih.gov>). Phyre2 server (11) was also applied for predicting the function and structure of uncharacterized gene products. Molecular phylogenetic analysis was performed using MEGA (12) by Maximum Likelihood method based on the Poisson correction model (13). Initial tree for the heuristic search were obtained automatically by applying the Maximum Parsimony method. All positions containing gaps and missing data were eliminated.

**Untargeted global metabolomic analysis**

Preparation of samples for metabolomics analysis and the number of replicates were similar to the procedure explained in transcriptomic analysis, except two types of media including soft agar BAPHK and BHIHK were applied. Within 24 to 30 hours after incubation, the agar layer was removed, and samples were harvested from the surface of polystyrene plates using phosphate-buffered saline (or PBS, pH 7.2). Similar procedure was undertaken for control preparation, except plates were not inoculated with bacterial cells. Cells were removed from collected samples by centrifugation at 4700 × g for 30 min at 4 °C followed by filtration using a Millex GP 0.22 μm filter with a PES membrane (Merck Millipore). Collected samples were freeze dried, weighted and subjected to further purification using a solvent system consisting of acetonitrile: methanol: acetone (8:1:1). Metabolomic analysis was conducted in Southeast Center for Integrated Metabolomics (SECIM) Center at the University of Florida. Extracts was pre-normalized prior to subjecting to global metabolomic analysis. Global metabolomics profiling was performed on a Thermo Q-Exactive Oribtrap mass spectrometer with Dionex UHPLC and autosampler. All samples were analyzed in positive and negative heated electrospray ionization with a mass resolution of 35,000 at m/z 200 as separate injections. Separation was achieved on an ACE 18-pfp 100 x 2.1 mm, 2 μm column with mobile phase A as 0.1% formic acid in water and mobile phase B as acetonitrile. The flow rate was 350 μl/min with a column temperature of 25°C. A 4 and 2 μl samples were injected for negative and positive ions, respectively. Data from positive and negative ion modes were separately subjected to statistical analyses and all subsequent data analyses were normalized to the sum of metabolites for each sample. The open-source software MZmine (14) was used to identify features and deisotopes and the MetaboAnalyst software (15) was applied for comparative and statistical analyses.

**References**

1. Moye ZD, Valiuskyte K, Dewhirst FE, Nichols FC, Davey ME. Synthesis of sphingolipids impacts survival of *Porphyromonas gingivalis* and the presentation of surface polysaccharides. Front Microbiol. 2016;7:1919.

2. Davey ME, Duncan MJ. Enhanced biofilm formation and loss of capsule synthesis: deletion of a putative glycosyltransferase in *Porphyromonas gingivalis*. J Bacteriol. 2006;188(15):5510-23.

3. Gardner RG, Russell JB, Wilson DB, Wang GR, Shoemaker NB. Use of a modified *Bacteroides*-*Prevotella* shuttle vector to transfer a reconstructed beta-1,4-D-endoglucanase gene into *Bacteroides uniformis* and *Prevotella ruminicola* B14. Appl Environ Microbiol. 1996;62(1):196-202.

4. Crocker JC, Grier DG. Methods of digital video microscopy for colloidal studies. Journal of Colloid and Interface Science. 1996;179(1):298-310.

5. Gibiansky ML, Conrad JC, Jin F, Gordon VD, Motto DA, Mathewson MA, et al. Bacteria use type IV pili to walk upright and detach from surfaces. Science. 2010;330(6001):197.

6. McClure R, Balasubramanian D, Sun Y, Bobrovskyy M, Sumby P, Genco CA, et al. Computational analysis of bacterial RNA-Seq data. Nucleic acids research. 2013;41(14):e140.

7. Powell D. Degust: Take the time to digest and appreciate your Differential Gene Expression data. <Http://victorian-bioinformaticsconsortium.github.io/degust/>. 2016.

8. Kanehisa M, Furumichi M, Tanabe M, Sato Y, Morishima K. KEGG: new perspectives on genomes, pathways, diseases and drugs. Nucleic Acids Res. 2017;45(D1):D353-D61.

9. Caspi R, Altman T, Billington R, Dreher K, Foerster H, Fulcher CA, et al. The MetaCyc database of metabolic pathways and enzymes and the BioCyc collection of Pathway/Genome Databases. Nucleic Acids Res. 2014;42(Database issue):D459-71.

10. Caspi R, Billington R, Ferrer L, Foerster H, Fulcher CA, Keseler IM, et al. The MetaCyc database of metabolic pathways and enzymes and the BioCyc collection of pathway/genome databases. Nucleic Acids Res. 2016;44(D1):D471-80.

11. Kelley LA, Mezulis S, Yates CM, Wass MN, Sternberg MJ. The Phyre2 web portal for protein modeling, prediction and analysis. Nat Protoc. 2015;10(6):845-58.

12. Kumar S, Stecher G, Tamura K. MEGA7: Molecular Evolutionary Genetics Analysis Version 7.0 for Bigger Datasets. Mol Biol Evol. 2016;33(7):1870-4.

13. Zuckerkandl E, Pauling L. Evolutionary divergence and convergence in proteins. In: Bryson V, Vogel HJ, editors. Evolving Genes and Proteins. New York: Academic Press; 1965. p. 97-166.

14. Pluskal T, Castillo S, Villar-Briones A, Oresic M. MZmine 2: modular framework for processing, visualizing, and analyzing mass spectrometry-based molecular profile data. BMC Bioinformatics. 2010;11:395.

15. Xia J, Wishart DS. Using MetaboAnalyst 3.0 for comprehensive metabolomics data analysis. Curr Protoc Bioinformatics. 2016;55:14.0.1-.0.91.
