# Supplementary material for: Amino acids as wetting agents: surface translocation by Porphyromonas gingivalis
Source: ISME J. 2019 Feb 19;13(6):1560–74. doi: 10.1038/s41396-019-0360-9 (PMC6775972; doi:10.1038/s41396-019-0360-9)
Supplement: Supplementary file 2 — Supplemental Figures and Video legends [file 41396_2019_360_MOESM2_ESM.docx]

**
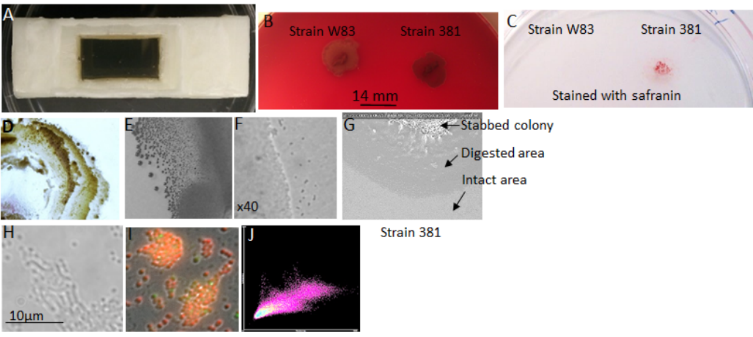
Supplemental Figures**

**Supplemental Fig 1.** Surface translocation and colonization by *P. gingivalis*. (A) The chamber slide designed for time-lapse microscopic examination. (B) comparison of two strains 381 and W83 colonizing the interface between polystyrene plate and soft agar. (C) Safranin staining after washing the polystyrene plate surface shows strain 381 displays surface attachment, but W83 does not. (D) Formation of zonal patterns after surface translocation and (E) satellite microcolonies formed at peripheral areas by strain 381; (F) Microscopic analysis of cells of strain 381 at peripheral area forming microcolonies. (G) Cryo-SEM image shows digestion of nutrients and medium by strain 381 at early stage of surface translocation, corresponding to hydration stage. (H) Elongated and motionless cells formed at late stage of surface translocation, while other individual cells displayed continuous back and forth movements, ultimately forming an aggregate of disordered cells. (I, J) Live/dead staining showed that the islands of misshaped and disordered cells contained numerous dead cells, but embedding a few viable cells; (J) colocalization intensity graph representing the green pixels (live cells) (vertical axis) versus the red pixels (dead cells) (horizontal axis).


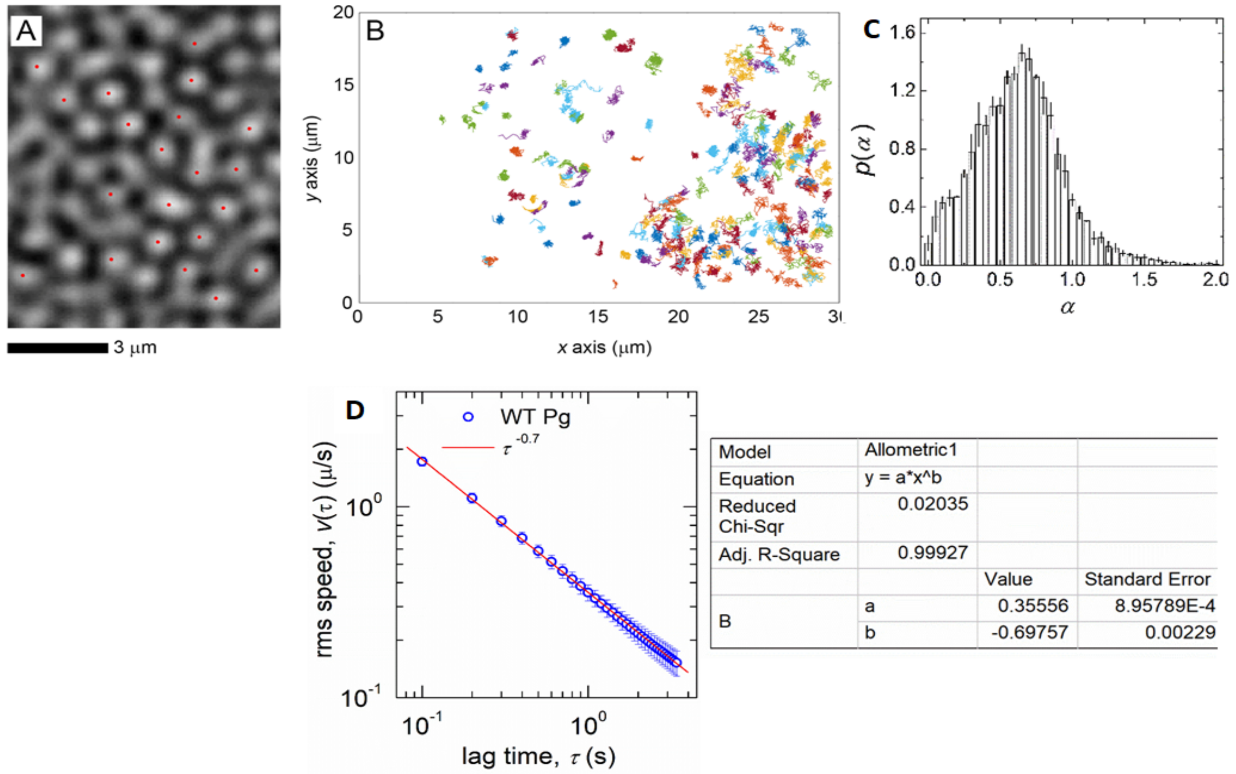


**Supplemental Fig 2.** Tracking of *P. gingivalis* cell movement. (A) To quantify the motion of individual cells between 50 to 85 hours of surface translocation in chamber slides, we identify cell locations and track their motion using the approach described in the Methods section. Here, we overlay a scatter plot of measured cell locations on the corresponding snapshot of *P. gingivalis* cells from video microscopy. Cells not identified in this figure were found to move too fast to be tracked frame-by-frame and are not included in our analysis. (B) To illustrate the overall shape and domain explored by tracked cells, we plot traces of all tracked cells within our field of view through a representative video (duration: 60s; frame rate: 10 Hz). Most tracked cells are observed to be confined by their neighbors while covering a domain of 1-2 microns, with some cells able to move around their neighbors, exhibiting more elongated tracks. (C) To ensure that apparent power-law in motility classification did not arise from ensemble averaging single cell tracks that exhibited more complicated motion, we measured *α* at all *τ* ’s for each cell by computing the logarithmic derivative of each single-cell Δ*r*^2^(*τ* ), which is given by
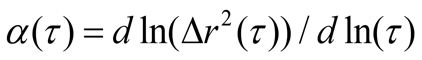
. Computing a probability density function of all measured *α*’s, we find a slightly skewed distribution that peaks at approximately *α* = 0.6. (D) Computing the root-mean-square (rms) speed from tracking of 10-20% of cell population displaying the slowest movement (see Method section) shows that the mean speed is about 2 µm/s at the shortest time-scales measured. By contrast, at the longest time-scales measured, the mean speed is about 0.15 µm/s. In our case, since the MSD scales like *τ* ^0.6^, the rms speed should scale like *τ* ^-0.7^ and, indeed, computing the rms speed from our tracks shows this scaling.


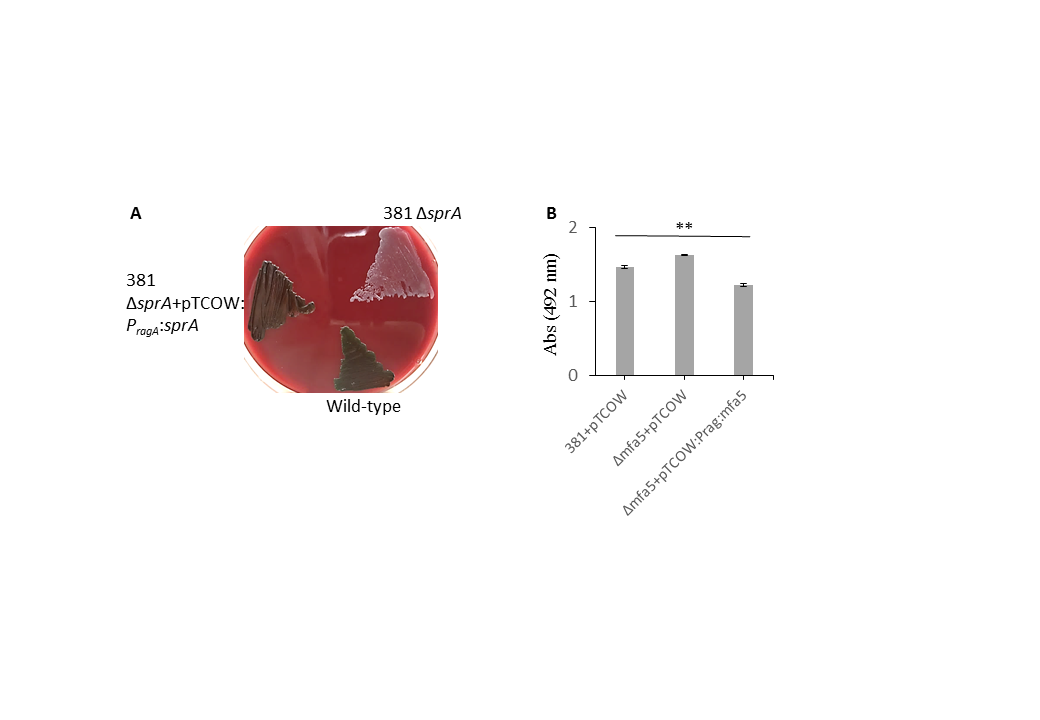


**Supplemental Fig 3.** Phenotypic characterization of *P. gingivalis* strain 381 mutants. (A) The ∆*sprA* mutant is defective in binding to heme, resulting in a non-pigmented phenotype on BAPHK medium. Heme binding was restored with *in trans* complementation. (B) Biofilm assessment indicates that Mfa5 protein negatively regulates biofilm formation.


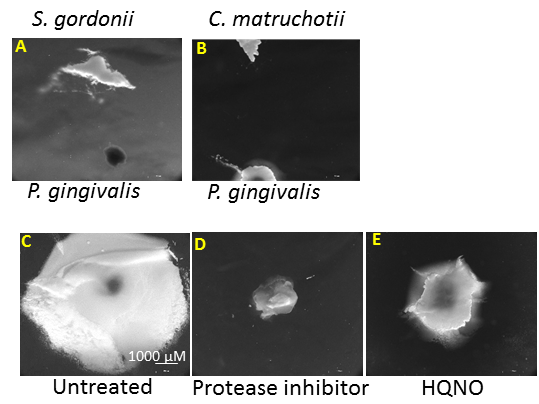


**Supplemental Fig 4.** Surface translocation by *P. gingivalis* is influenced by different oral bacteria or chemical substances. (A) Co-culture of *P. gingivalis* strain 381 with *S. gordonii* suppressed surface translocation, while *C. matruchotii* did not (B). (C) surface translocation by *P. gingivalis* over 95 hours in an untreated chamber slide; (D) treated with 1x Clontech’s ProteoGuard EDTA-Free Protease Inhibitor Cocktail; (E) treated with 2-*n*-heptyl-4-hydroxyquinoline *N*-oxide (HQNO) (10 µg/ml). Images were captured using 2x magnification of an inverted Nikon Eclipse Ti microscope (Nikon, Tokyo, Japan).


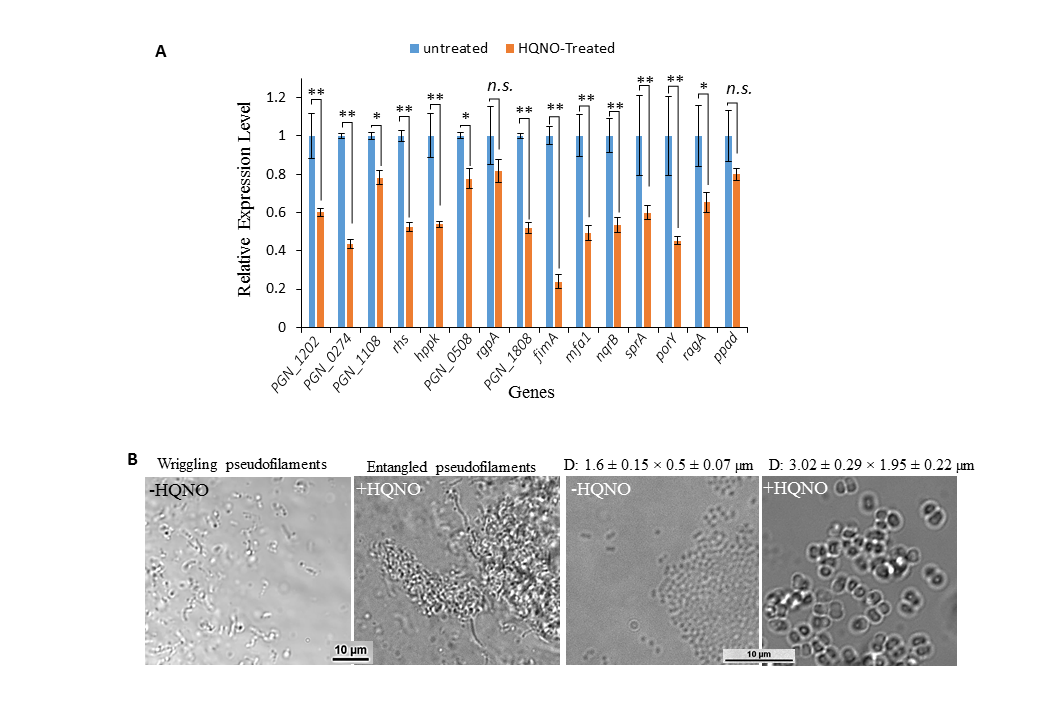
**Supplemental Fig 5.** Impact of 2-*n*-heptyl-4-hydroxyquinoline *N*-oxide as a specific inhibitor of the Na^+^-NQR function and quinone oxidation on gene expression and surface translocation. (A) qRT-PCR analysis indicates that HQNO (10 µg/ml) (in BAPHK 0.3% agar) significantly reduced the expression of a variety genes which were upregulated or highly expressed during surface translocation. (B) Comparison of *P. gingivalis* in the chamber slide untreated and treated with HQNO. HQNO treatment caused pseudofilaments in disordered status with defective wriggling and some cells turned into giant phenotype while they proliferate in cell clusters. Cell sizes at top of each image are the average of the size of 20 cells. The data in histogram represent the means ± STD error for at least four independent repetitions, and asterisks indicate pairs of significantly different values (*post hoc* Tukey's HSD test: *, *P* < 0.05; **, *P* < 0.01; n.s., not significant). D: dimension.

**Supplemental Video Legends**

**Supplemental Video 1.** Time-lapse microscopy recordings of *P. gingivalis* forming wriggling pseudofilaments. Video length: 1min 30 sec; interval: 15 mSec; frame rate: 29 frames/sec.

**Supplemental Video 2.** Time-lapse microscopy recordings of *P. gingivalis* surface translocation and outward spreading from stabbed site within 36 hours. Original file was compressed by merging every 20^th^ frames then sped up 32 times. Video length: 33 sec; interval: 5 min; frame rate: 29 frames/sec.

**Supplemental Video 3.** Time-lapse microscopy recordings of super-diffusive cell-driven motility by *P. gingivalis* about 85 hours into surface translocation course. Video length: 1min 30 sec; interval: 15 mSec; frame rate: 29 frames/sec.

**Supplemental Video 4.** Time-lapse microscopy recordings of surface attachment of motile cells and biofilm formation and development by *P. gingivalis*. Video length: 1min; interval: 15 mSec; frame rate: 29 frames/sec.

**Supplemental Video 5.** Time-lapse microscopy recordings of passive fluorospheres which did not display motion under the same condition. Video length: 30 sec; interval: 15 mSec; frame rate: 29 frames/sec.

**Supplemental Video 6.** Time-lapse microscopy recordings of antibiotic-treated cells about 85 hours after treatment which did not display motion under the same condition. Video length: 10 sec; interval: 15 mSec; frame rate: 29 frames/sec.

**Supplemental Video 7.** Time-lapse microscopy recordings of non-motile species *P. intermedia*,  *C. matruchotii*, and *S. gordonii* at about 85 hours of incubation which did not show movement under the same growth condition. Video length: 12 sec; interval: 15 mSec; frame rate: 29 frames/sec.

**Supplemental Video 8.** Time-lapse microscopy recordings of *P. gingivalis* mutants including during ∆*sprA* and ∆*mfa5*. Jelly-like texture formed by *P. gingivalis* ∆*sprA*, and abolished surface translocation for both mutants. Video length: 20 sec; interval: 15 mSec; frame rate: 29 frames/sec.

**Supplemental Video 9.** Time-lapse microscopy recordings of *P. gingivalis* ∆*fimC*. This video shows emerging pseudofilaments of ∆*fimC* mutant defective in wriggling motion, migrating cells on surface which is similar to sub-diffusive cell-driven motility of the wild-type, and a swarming-like social spreading motility on the surface unable to transition to surface attachment. Video length: 40 sec; interval: 15 mSec; frame rate: 29 frames/sec.
