# Supplementary material for: Amino acids as wetting agents: surface translocation by Porphyromonas gingivalis
Source: ISME J. 2019 Feb 19;13(6):1560–74. doi: 10.1038/s41396-019-0360-9 (PMC6775972; doi:10.1038/s41396-019-0360-9)
Supplement: Supplementary file 12 — Supplemental Table-1 [file 41396_2019_360_MOESM12_ESM.docx]

| **List of primers** |
| --- |
| **qRT-PCR primers** |
| CCAAGTCGCGTGAAGGAAGA,q16sRNA.Fw |
| CACCGCTGACTTACCGAACA,q16sRNA.Rv |
| TTCGGGGCAACACTCGTAAA,qFimA.Fw |
| CCAAGTCGGCTCCGTTTTTC,qFimA.Rv |
| GGAGAACGTCGCCAATACCT,qMfa1.Fw |
| AATCAGCCAAAGTCGGCACT,qMfa1.Rv |
| ACTGACGCTGATTCGCAAGA,qPGN.832.Fw |
| ACGGAAGCTGACACCGAAAT,qPGN.832.Rv |
| GGCTGAGTGACCTCCGATTC,qPGN.293.Fw |
| CGAGAGCCTGGTGGTTGTAG,qPGN.293.Rv |
| TGAGGTCATCGGCATAGCAC,qPGN.2001.Fw |
| TTTGCCAGAGACAAGCCCAA,qPGN.2001.Rv |
| TCGGCGAACCTGATCCTTTC,qPGN.898.Fw |
| AGACACTCGTCCCTGCTACT,qPGN.898.Rv |
| CGTGGAAGAAACGCTGTCGG,qPGN.1818.Fw |
| AATCCGTTTCCTCCGGCCTT,qPGN.1818.Rv |
| CCGAACTAACACATTATCCCGGC,qPGN.1819.Fw |
| CTGTCTCCAGTCCGACAATGA,qPGN.1819.Rv |
| CGGAAATTCCAAAGGATTTGCT,qPGN.1820.Fw |
| AAATTACGGTCAGTGGAGCG,qPGN.1820.Rv |
| TGACTGACGAAGCGATCGAAGA,qPGN.1052.Fw |
| GCGGACACAGTTGTTACAGGG,qPGN.1052.Rv |
| CGCTCAGCAAGAAGGAACGC,qPGN.508.Fw |
| GAGCACCTTGGCCGGTACAT,qPGN.508.Rv |
| AGGTGGGTTCGTCAGTCATGG,qPGN.1808.Fw |
| CCGAAACAGGCGGAACAATCG,qPGN.1808.Rv |
| TTCCTGACGGAGTGGCAGAC,qPGN.1970.Fw |
| CGTAAGACTTGCCGTCAACGA,qPGN.1970.Rv |
| GGCATTGGACTCGTTCCGTT,qPGN.228.Fw |
| CGACTCCCATTGCACCGTTA,qPGN.228.Rv |
| GGCTGCCTTACGGGCAATTA,qPGN.1474.Fw |
| TCAGGTTCTCCTCCGTCGCT,qPGN.1474.Rv |
| GTACGATCTCTCGGGTGAGCA,qPGN.1202.Fw |
| CAACGGTACGACGAGCCAGT,qPGN.1202.Rv |
| GATGGATCACTACCGCCGGG,qPGN.274.Fw |
| CCTCCCAGTAGCGCATTCTCA,qPGN.274.Rv |
| TATCTGCTCGACAACCTCACGG,qPGN.1108.Fw |
| GCATGGTGGTACGATGCCTTG,qPGN.1108.Rv |
| GCGGTTTGGTAGGAGCCTCA,qPGN.115.Fw |
| CCCGGATTGAGCACTCGGAT,qPGN.115.Rv |
| Upstream fimC (PGN_0183).Fw  TTTCGCCTGACCGTCAGGGCTTGG |
| Upstream fimC (PGN_0183).Rv  TGTAGATAAATTATTAGGTATACTACTGACAGCTTCAATAGCTGTCGGATTAGTATTCTG |
| Downstream fimC (PGN_0183).Fw  ACCGATGAGCAAAAAAGCAATAGCGGAAGCGATCGGGCGGATTATTCATTATGAGAACAA |
| Downstream fimC (PGN_0183).Rv  GACCTCTAAATACTGTACATCAGG |
| LinM3(U.D.PG809.Erm).Fw  AAGCCGTGAAGGGCATCGGTCTGA |
| LinM3(U.D.PG809.Erm).Rv  CAGTTCTTCATCGGGGAGGATGAT |
| LinM4(U.D.PGN291.Erm).Fw  ATGAAAGGAATCAAGGAAGTGCTGC |
| LinM4(U.D.PGN291.Erm).Rv  GCCATAGCCCACCCAATGCTC |
| LinM5(U.D.PGN832.Erm).Fw  CGCTCCTTTTATGCCAAGAACTCCTC |
| LinM5(U.D.PGN832.Erm).Rv  GAGCATTCTGTTCGGCTCTTACCC |
| **Primer for confirming knockouts** |
| Nter.Erm.Rv.General  gattacttatatttgcttgtcttat  Cter.Erm.Fw.General  aaaccttaatgctcaaattgtttgt  M4Fw.UpXout.PGN0291  tcctgttcaaagaacaatcctagcgagc  M4Rv.DownXout.PGN0291  agcaccgatcgtggtgtttccgacaacc  M5Fw.UpXout.PGN0832  gtaggataaggcataaccacccttgagg  M5Rv.DownXout.PGN0832  tcatcggtggcatcggatctcatataac  M3Fw.UpXout.PG0809  caccacttattctgcctatcaggggaag  M3Rv.DownXout.PG0809  gccgatggatcggtgtcttgctcgatcc |
| **Primers for complementations** |
| \| GGTGGTAAGCTTccactttattccataaaatcgaacc,P.ragA.HindIII.Fw \| \| --- \| \| GGTGGTGCTAGCagacttttcttttgcgttaaact,P.ragA.NheI.Rv \| \| GGTGGTGCTAGCATGAAAAGAATGACGCTATTCT,RagA ORF381.NheI.Fw \| \| GGTGGTGCATGCTTAGAAAGAAATCTGAATACCACC,RagA ORF381.SphI.Rv \| \| GGTGGT GCATGCTTAGTGGTGATGGTGATGATGGAAAGAAATCTGAATACCACCT,RagA6xHisvORF381.SphI.Rv \| \| gaggatattgacgcttatttcgataCCACTTTATTCCATAAAATCGAAC,P.ragA.Fw.AsmC1 \| \| tagtattctgcatAGACTTTTCTTTTGCGTTAAACTTAAAATTATTAC,P.ragA.Rv.AsmC1 \| \| aaaagaaaagtctATGCAGAATACTAATCCGACAGCTATTATG,PGN183.Fw.AsmC1 \| \| gcaccgccgccgcaaggaatggtgcatgTTAAAGTCCTTGTTGTATGATCTTAATATCG,PGN183.Rv.AsmC1 \| \| gaggatattgacgcttatttcgataCCACTTTATTCCATAAAATCGAAC,P.ragA.Fw.AsmC2 \| \| atcgtttcatcatAGACTTTTCTTTTGCGTTAAACTTAAAATTATTAC,P.ragA.Rv.AsmC2 \| \| aaaagaaaagtctATGATGAAACGATATACAATAATTC,PGN291.Fw.AsmC2 \| \| gcaccgccgccgcaaggaatggtgcatgTTAGTTGACTACAACTTTCCTTAC,PGN291.Rv.AsmC2 \| \| gaggatattgacgcttatttcgataCCACTTTATTCCATAAAATCGAAC,P.ragA.FW.AsmC3 \| \| tgtttttcttttccatAGACTTTTCTTTTGCGTTAAACTTAAAATTATTAC,P.ragA.Rv.AsmC3 \| \| aaaagaaaagtctATGGAAAAGAAAAACAAACCCATTC,PGN832.Fw.AsmC3 \| \| gcaccgccgccgcaaggaatggtgcatgCTACTGCGTCAGATTGAAACGGAAG,PGN832.Rv.AsmC3 \| |
