# Supplementary material for: Amino acids as wetting agents: surface translocation by Porphyromonas gingivalis
Source: ISME J. 2019 Feb 19;13(6):1560–74. doi: 10.1038/s41396-019-0360-9 (PMC6775972; doi:10.1038/s41396-019-0360-9)
Supplement: Supplementary file 13 — Supplemental Table-2 [file 41396_2019_360_MOESM13_ESM.docx]

| **Supplemental Table 2. RNA-seq data and differential gene expression of the genes for the cells**  **grown at the subsurface of soft agar versus biofilm cells** | | | | |
| --- | --- | --- | --- | --- |
|  | | | | |
| **Gene number in two strains (homology percentage)** | **Predicted molecule/family** | **Predicted function/pathway** | **Fold change in 381**  **(*q*-value < 0.01, unless otherwise stated)** | **Fold change in W83**  **(*q*-value < 0.01, unless otherwise stated)** |
| **Membrane associated pathways** | | | | |
| PGN_1816  PG01889 (70%) | Unknown/ lipoprotein | unknown | 3.5 | 3.6 |
| PGN_1817  PG01890 (87%) | Immunoreactive 84kd antigen pg93 | Transport protein | 16.6 | 4.5 |
| PGN_1818  Absent in W83 | Rhs-repeat containing from ABC toxin complex/ Membrane protein | Toxin | 32.3 | - |
| PGN_1819  Absent in W83 | RpoE2-like |  | 50.4 | - |
| PGN_1820  PG01891(94%) | Unknown/Membrane protein |  | 48.2 | 4.9 |
| PGN_1821  PG01892 (98%) | Rhs-repeat containing from ABC toxin complex/ Membrane protein | Toxin | 14.9 | 3.7 |
| PGN_1822  Absent in W83 | Unknown | Unknown | 114 | - |
| PGN_1823  PG01893 (99%) | TPR-containing domain/N-terminal acetyltransferase-like protein | Protein-protein interaction/ within N-acetyl transferase complexes | 14.3 | 5.1 |
| PGN_1824  Absent in W83 | Rhs-repeat containing from ABC toxin complex/ Membrane protein | Toxin | 6.5 | - |
| PGN_1050  PG0889 (99%) | Unknown | Unknown | 12.5 | 6.4 |
| PGN_1051  PG0887 | Unknown | Unknown | 40.2 | - |
| PGN_1052  PG0886 (99%) | 7,8-Dihydro-6-hydroxymethylpterin-pyrophosphokinase, HPPK | Folic acid and derivative biosynthetic process | 32 | 8.3 |
| PGN_1053  PG0885 (100%) | putative phospho-2-dehydro-3-deoxyheptonate aldolase/chorismate mutase | Probably maintaining the balance of aromatic amino acids in the cell | 10.4 | 4.5 |
| PGN_1054  PG0884 (98%) | Glycosyltransferase/VimF | Virulence modulating | insignificant | 4.5 |
| PGN_1055  PG0883(99%) | putative polysaccharide deacetylase/ VimE | Virulence modulating | 2 | 1.3 |
| PGN_0508  PG1605 | aminopeptidase | bleomycin hydrolase-like | 28 | 7.1 |
| PGN_0509  PG1604 | immunoreactive 84 kDa antigen |  | 7.1 | 2.5 |
| PGN_0510  PG1603 | probable xanthosine triphosphate pyrophosphatase |  | 7.2 | 2.8 |
| PGN_1905  PG1974 | Unique lipoprotein |  | 27 | 15.5 |
| PGN_1806  PG1879 | Putative patatin-like phospholipase/glycoprotein | Probable effector molecules to target host cellular | 11.95 | 3 |
| PGN_1807  PG1880 | Glycosyltransferase | Unknown | 15.25 | 3.8 |
| PGN_1808  PG1881 (98%) | Fimbrilin | unknown | 23.5 | 5.4 |
| PGN_1809  Next to PG1881 | Unknown | Unknown | 16.2 | - |
| PGN_1928  PG1983 (86%) | CRISPR-associated protein |  | 12.5 | 4.7 |
| PGN_1929  PG1984 (80%) | CRISPR-associated protein |  | 15.8 | 5.5 |
| PGN_1930  PG1985 (94%) | CRISPR-associated protein |  | 16 | 5.5 |
| PGN_1931  PG1986 (89%) | CRISPR-associated protein |  | 17.3 | 6.2 |
| PGN_1932  PG1987 (92%) | CRISPR-associated protein |  |  |  |
| PGN_1933  PG1988 (47%) | CRISPR-associated protein |  | 23.2 | 5.6 |
| PGN_1934  PG1989 (61%) | Unknown | Unknown |  |  |
| PGN_1935  PG_RS08780 (98%) | Putative Mg transporter/ membrane protein | Unknown | 6.6 | 5.7 |
| PGN_0184  PG2135 (43%) | FimD/lipoprotein | Major fimbrial subunit | 7 | 1.6 |
| PGN_0185  PG2136 (43%) | FimE | Major fimbrial subunit | 6 | 0.7 |
| PGN_0444  PG1667 | Outer membrane efflux protein/TolC family |  | 0.1 | 0.5 |
| PGN_0445  PG1666 | Membrane fusion protein of efflux RND transporter/ periplasmic adaptor subunit |  | 0.1 | 0.6 |
| PGN_0446  PG1665 | ABC transporter ATP-binding protein |  | 0.1 | 0.6 |
| PGN_0447  PG1664 | ABC transporter ATP-binding protein |  | 0.1 | 0.6 |
| PGN_0448  PG1663 | ABC transporter ATP-binding protein/putative Spermidine/putrescine/amino acid ABC transporter |  | 0.1 | 0.6 |
| PGN_0715  PG0679 | Outer membrane efflux protein/TolC family |  | 0.2 | 0.5 |
| PGN_1539  PG0540 (29%) | Outer membrane efflux protein /TolC family |  | 8.7 | insignificant |
| PGN_1540  PG0285 (23%) | ABC transport membrane efflux protein/AcrB/D/F family |  | 6 | 0.2 |
| PGN_2012  PG0063 | Outer membrane efflux protein/TolC family |  | 10 | insignificant |
| PGN_2013  PG0064 | Cation/multidrug efflux system protein |  | 8.8 | insignificant |
| PGN_2014  PG0065 | Efflux RND transporter periplasmic membrane fusion protein |  | 9.1875 | insignificant |
| PGN_0206  PG2162 | lipid A disaccharide synthase |  | 6.9 | 5.6 |
| (PGN_0679)  PG0638 | tetraacyldisaccharide 4'-kinase (LpxK) |  | 6.5 | insignificant |
| PGN_1203  PG1106 | UDP-N-acetylmuramoylalanyl-D-glutamyl-2, 6-diaminopimelate--D-alanyl-D-alanyl ligase (MurF) |  | 2.8 | 0.36 |
| **T9SS and its cargo associates/cognate regulatory genes** | | | | |
| PGN_0832  PG0809 | SprA | T9SS component | 7.4 | insignificant |
| PGN_1673  PG0291 | PorN | T9SS component | 3 | insignificant |
| PGN_1674  PG0290 | PorM | T9SS component | 6.4 | insignificant |
| PGN_1675  PG0289 | PorL | T9SS component | 7.3 | 1.3 |
| PGN_1676  PG0288 | PorK | T9SS component | 11.1 | insignificant |
| PGN_1677  PG0287 | PorP | T9SS component | 8.06 | insignificant |
| PGN_1019  PG0928 | PorX | T9SS component | 0.11 | 0.4 |
| PGN_1877  PG1947 | PorW | T9SS component | 3.47 | 0.67 |
| PGN_2001  PG0052 | PorY | T9SS component | insignificant | insignificant |
| PGN_0778  PG0751 | PorT | T9SS component | insignificant | insignificant |
| PGN_0022  PG0026 | PorU | T9SS component | 2.49 | 1.15 |
| PGN_0023  PG0027 | PorV | T9SS component | 5.5 | insignificant |
| PGN_0645  PG0602 | PorQ | T9SS component | insignificant | insignificant |
| PGN_0274  PG0162 | RNA polymerase sigma-e factor ECF subfamily |  | 3.2 | insignificant |
| PGN_1970  PG2024 (99%) | RgpA gingipain | Proteolysis/virulence/ secreted by T9SS | 22.6 | 2.97 |
| PGN_1466  PG0506 | RgpB gingipain | Proteolysis/virulence/ secreted by T9SS | 12.5 | 2.1 |
| PGN_1728  PG1844 (90%) | Kgp gingipain | Proteolysis/virulence/ secreted by T9SS | 11 | ND |
| PGN_0898  PG1424 | PPAD (peptidylarginine deiminase) | Protein-citrullinating/  secreted by T9SS | 13.4 | 5.2 |
| **Nutrient transport pathway and cognate regulatory genes** | | | | |
| PGN_0557  PG1552 | HmuR/TonB-dependent receptor | Hemin binding | 0.25 | insignificant |
| PGN_0558  PG1551 | HmuY protein-heme binding | Heme binding | 0.8 | 15.6 |
| PGN_0683  Absent in W83 | TonB-linked receptor Tlr | Hemin binding | 14.5 | - |
| PGN_1474  PG0498 | S-Ribosylhomocysteinase (LuxS) | Autoinducer production | 5.7 | 4 |
| PGN_0809  PG0785 | Putative TonB | Nutrient transport | 2 | insignificant |
| PGN_0806  PGN0872 | Putative MotA/TolQ/ExbB proton channel protein | Nutrient transport/energy provider | 6 | 3.7 |
| **Metabolic pathways** | | | | |
| PGN_0634  PG0588 | 3-methyl-2-oxobutanoate hydroxymethyltransferase (panB) |  | 3.536585 | 3.129 |
| PGN_1204  PG1114 | aspartate alpha-decarboxylase  PanD |  | insignificant | 5.122 |
| PGN_1206  PG1116 | bifunctional 5,10-methylene-tetrahydrofolate dehydrogenase/ 5,10-methylene-tetrahydrofolate cyclohydrolase (FolD) |  | 3 | insignificant |
| PGN_0667  PG0625 | GTP cyclohydrolase I (FolE) |  | 6.1 | 2.1 |
| PGN_2006  PG0057 | Nicotinate phosphoribosyltransferase (pncB) |  | 6 | insignificant |
| PGN_0678  PG0637 | Thiamine monophosphate kinase (thiL) |  | 6 | insignificant |
| PGN_0643  PG0599 | Putative 3,4-dihydroxy-2-butanone 4-phosphate synthase (ribBA) |  | 3.2 | insignificant |
| PGN_0267  PG0155 | putative riboflavin biosynthesis protein (ribD) |  | 1.7 | insignificant |
| PGN_0757  PG0725 | Putative 5-amino-6-(5-phospho-D-ribitylamino)uracil phosphatase |  | 3.7 | insignificant |
| PGN_0901  PG1428 | putative riboflavin synthase beta subunit (ribH) |  | 10.1 | 6.7 |
| PGN_0764  PG0733 | putative riboflavin synthase alpha subunit (ribE) |  | 2.8 | insignificant |
| PGN_0992  PG0957 | Putative riboflavin kinase / FMN adenylyltransferase (ribF) |  | 7.2 | 3.3 |
| PGN_1444  PG0529 | carbamoyl phosphate synthase small subunit (CarA) |  | 6 | 0.9 |
| PGN_1443  PG0530 | carbamoyl-phosphate synthase large subunit (CarB) |  | 6.2 | 0.5 |
| PGN_1827  PG1896 | S-adenosylmethionine synthetase |  | 0.6 | 0.24 |
| **Bioenergetic pathway** | | | | |
| PGN_0800  PG0776 | electron transfer flavoprotein alpha subunit (etfA-1) | Electron bifurcation | 1.46 | 3.4 |
| PGN_0801  PG0777 | electron transfer flavoprotein beta subunit (etfB-1) | Electron bifurcation | 2 | 4 |
| PGN_1173  PG1077 | electron transfer flavoprotein beta subunit (etfB-2) | Electron bifurcation | 12 | 6.4 |
| PGN_1174  PG1078 | electron transfer flavoprotein alpha subunit (etfA-2) | Electron bifurcation | 9.3 | 4.4 |
| PGN_1175  PG1079 | enoyl-CoA hydratase | Electron bifurcation | 10.5 | 3.1 |
| PGN_1176  PG1080 | 3-hydroxybutyryl-CoA dehydrogenase | Electron bifurcation | 9.2 | 2.7 |
| PGN_1654  PG0308 | electron transport complex RnfABCDGE type A subunit | Electron bifurcation | 2.5 | 3.1 |
| PGN_1658  PG0304 | electron transport complex RnfABCDGE type C subunit | Electron bifurcation | 3.2 | 3.1 |
| PGN_1657  PG0305 | electron transport complex RnfABCDGE type D subunit | Electron bifurcation | 3 | 2.8 |
| PGN_1656  PG0306 | electron transport complex RnfABCDGE type G subunit | Electron bifurcation | 2.7 | 2.8 |
| PGN_1367  PG1232 | glutamate dehydrogenase |  | 11 | 3.6 |
| PGN_1756  PG1809 | 2-oxoglutarate oxidoreductase subunit gamma |  | 6.5 | 6 |
| PGN_1755  PG1810 | 2-oxoglutarate oxidoreductase subunit beta |  | 5.5 | 5.4 |
| PGN_1753  PG1812 | 2-ketoisovalerate ferredoxin reductase |  | 6 | 4.7 |
| PGN_1752  PG1813 | ferredoxin, 4Fe-4S | Electron bifurcation | 18.3 | 6.6 |
| PGN_0723  PG0687 | CoA-dependent succinyl-CoA reductase |  | 16.6 | 18.6 |
| PGN_0724 PG0689 | 4-hydroxybutyrate dehydrogenase |  | 11.5 | 8 |
| PGN_0725 PG0690 | 4-hydroxybutyrate CoA-transferase |  | 8.1 | 7 |
| PGN_0727  PG0692 | 4-hydroxybutyryl-CoA dehydratase |  | 2.3  (*q*-value < 0.05) | insignificant |
| PGN_1179  PG1082 | Putative phosphate acetyltransferase (Pta) |  | 3.8 | 3.8 |
| PGN_0826  PG0802 | dihydrolipoamide dehydrogenase |  | 3 | 2.7 |
| PGN_0116  PG2180 | Na(+)-translocating NADH-quinone reductase subunit C | Chemiosmosis | 5.3 | 3.3 |
| PGN_0117  PG2179 | Na(+)-translocating NADH-quinone reductase subunit D | Chemiosmosis | 5.8 | 3.6 |
| PGN_0118  PG2178 | Na(+)-translocating NADH-quinone reductase subunit E | Chemiosmosis | 5.3 | 3.4 |
| PGN_1104  PG1314 | Chorismate synthase | pABA synthesis | 0.02 | 0.16 |
| PGN_1638  PG0324 | Histidine ammonia-lyase | Folate biosynthesis | 10.4 | 4.5 |
| PGN_1633  PGN_0329 | formiminotransferase-cyclodeaminase | Folate biosynthesis | 3 | 2.7 |
| PGN_1637  PG0325 | methenyltetrahydrofolate cyclohydrolase | Folate biosynthesis | 13.7 | 6.1 |
| PGN_1787  PG1854 | 5-formyltetrahydrofolate cyclo-ligase | Folate biosynthesis | 8.2 | 7.6 |
| PGN_0667  PG0625 | GTP cyclohydrolase I (FolE) | Folate biosynthesis | 6.1 | insignificant |
| PGN_1049  PG0890 | alkaline phosphatase |  | 10 | 5.2 |
| **Lactate metabolism** | | | | |
| PGN_1128  PG1340 | lactate permease |  | 2.6 | 2.5  (*q*-value < 0.06) |
| PGN_1268  PG1171 | Putative lactate dehydrogenase, Fe-S oxidoreductase subunit |  | 2.8 | 1.5  ( *q*-value < 0.04) |
| PGN_1269  PG1172 | Putative lactate dehydrogenase, Fe-S oxidoreductase subunit |  | 2.8 | 1.3  ( *q*-value < 0.02) |
| PGN_1270  PG1173 | Putative lactate dehydrogenase |  | 3 | 1.5 |
| PGN_1178  PG1081 | Putative acetate kinase |  | 2.2 | 2.7  ( *q*-value < 0.04) |
| **The extracytoplasmic function (ECF) sigma factors** | | | | |
| PGN_0450  PG1660 | RNA polymerase sigma-70 factor ECF subfamily |  | 0.4 | 1 |
| PGN_0970  PG0985 | RNA polymerase sigma-70 factor ECF subfamily |  | 0.03 | 0.08 |
| PGN_0638  PG0594 | RNA polymerase sigma factor RpoD (rpoD) |  | 1.0 | insignificant |
| PGN_0319 PG0214 | RNA polymerase sigma-70 factor ECF subfamily |  | 0.1 | 0.3 |
| PGN_1740 PG1827 | RNA polymerase ECF-type sigma factor |  | 1.0 | 0.9 |
| PGN_0274 PG0162 | RNA polymerase sigma-70 factor ECF subfamily |  | 3.1 | 1.5 |
| PGN_1202  PG1105 | RNA polymerase sigma-54 factor (rpoN) |  | 2 | 0.18 |
| PGN_1108  PG1318 | RNA polymerase ECF-type sigma factor |  | 3.1 | 2.3 |
| **Others** | | | | |
| PGN_0525  PG1586 | aerotolerance-related exported protein BatE |  | 1.3 | 2.9 |
| PGN_0526  PG1585 | aerotolerance-related exported protein BatD |  | 0.41 | 1.5 |
| PGN_0527  PG1584 | aerotolerance-related exported protein BatC |  | 0.2 | 1.2 |
| PGN_0528  PG1583 | aerotolerance-related exported protein BatB |  | 0.2 | 1.3 |
| PGN_0529  PG1582 | aerotolerance-related membrane protein BatA |  | 0.1 | 0.7 |
| PGN_0530  PG1581 | hypothetical protein |  | 0.09 | 0.6 |
| PGN_0531  PG1580 | von Willebrand factor A |  | 0.07 | 0.4 |
| PGN_0532  PG1579 | magnesium chelatase subunit I (MoxR) |  | 0.08 | 0.5 |
| PGN_1160, PGN_0839  PG0865, PG2176, PG0277,PG1350, PG1746 | transposase in ISPg2 |  | ~3.0 | ~3.0 |
