# Supplementary figures and images for: Amino acids as wetting agents: surface translocation by Porphyromonas gingivalis
Source: ISME J. 2019 Feb 19;13(6):1560–74. doi: 10.1038/s41396-019-0360-9 (PMC6775972; doi:10.1038/s41396-019-0360-9)

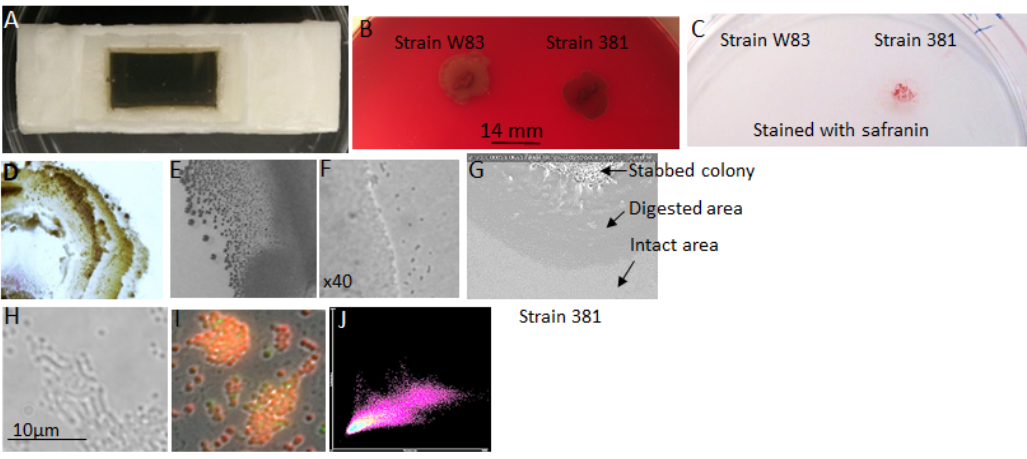

Supplement: Supplementary file 14 — Supplemental figure 1 [file 41396_2019_360_MOESM14_ESM.tif]

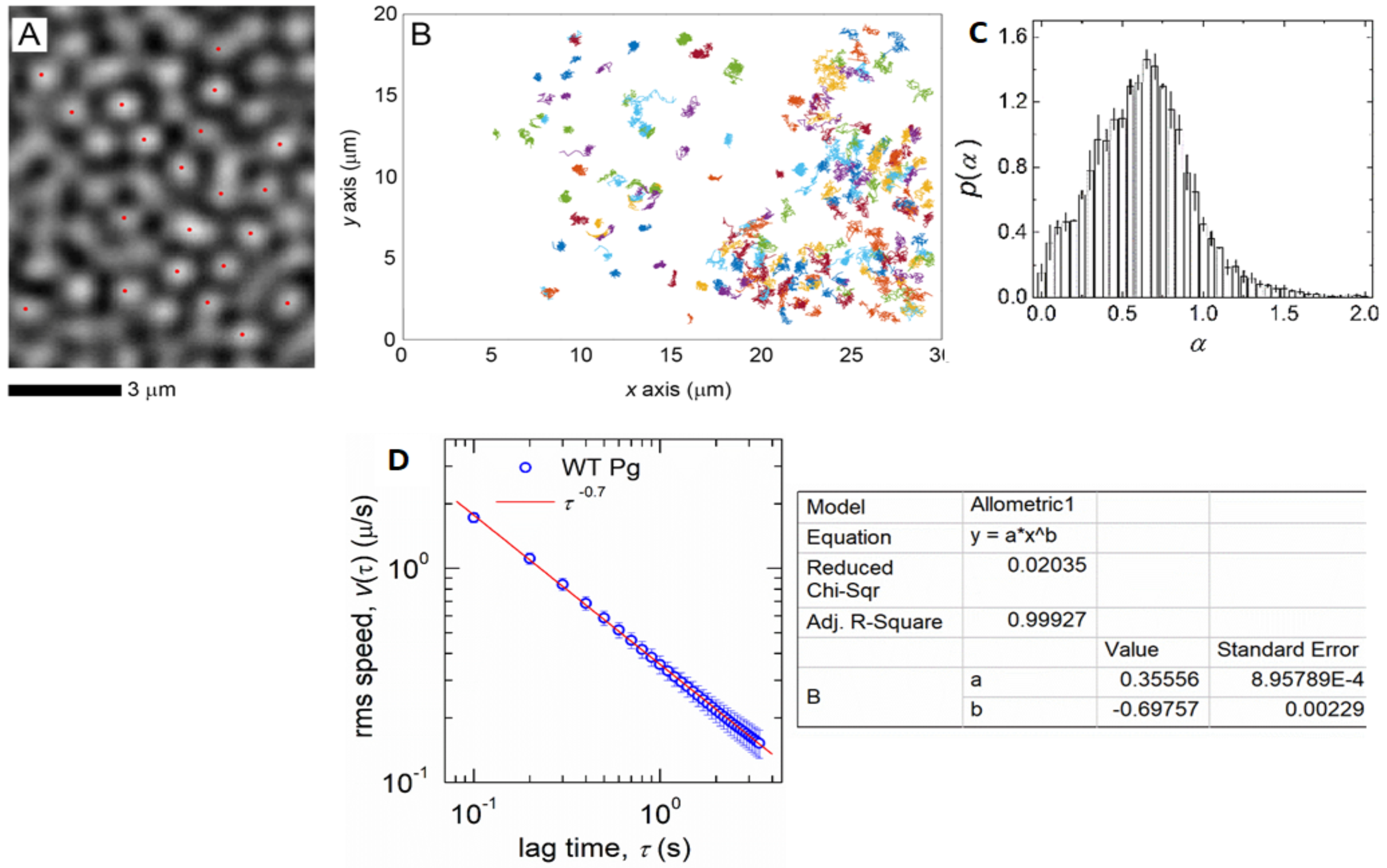

Supplement: Supplementary file 15 — Supplemental Figure 2 [file 41396_2019_360_MOESM15_ESM.tif]

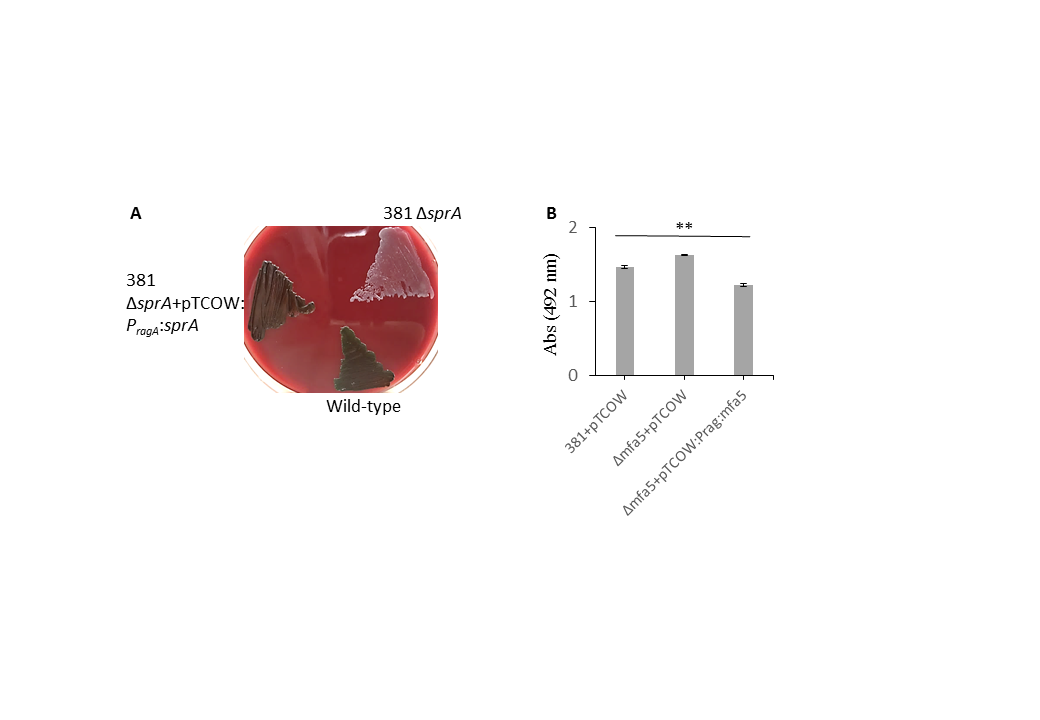

Supplement: Supplementary file 16 — Supplemental Figure 3 [file 41396_2019_360_MOESM16_ESM.tif]

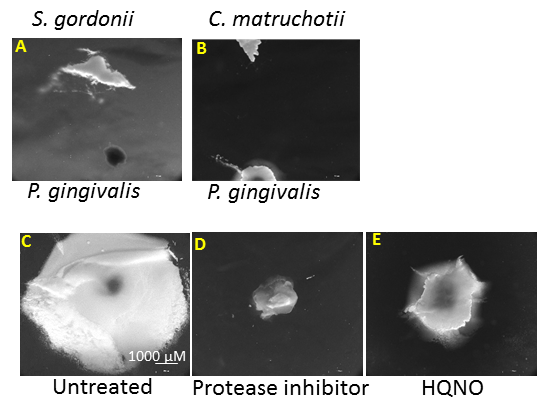

Supplement: Supplementary file 17 — Supplemental Figure 4 [file 41396_2019_360_MOESM17_ESM.tif]

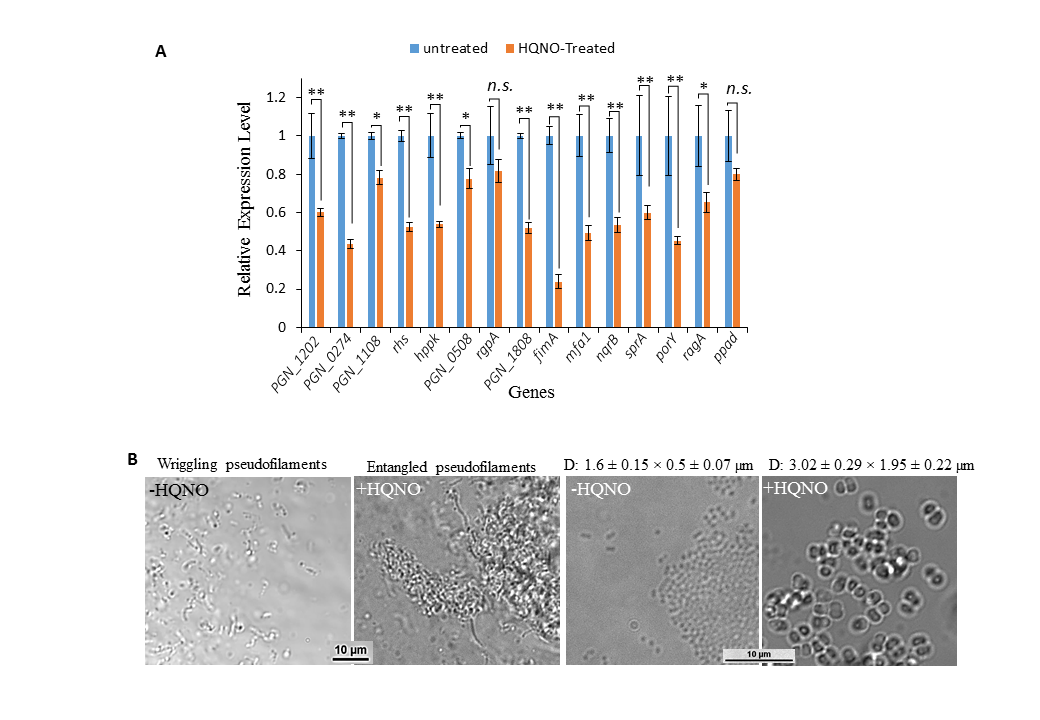

Supplement: Supplementary file 18 — Supplemental Figure 5 [file 41396_2019_360_MOESM18_ESM.tif]
